# Coordination Polymers of Vanadium and Selected Metal Ions with *N*,*O*-Donor Schiff Base Ligands—Synthesis, Crystal Structure, and Application

**DOI:** 10.3390/molecules30051104

**Published:** 2025-02-27

**Authors:** Dariusz Osypiuk, Agata Bartyzel, Beata Cristóvão

**Affiliations:** Department of General and Coordination Chemistry and Crystallography, Institute of Chemical Sciences, Faculty of Chemistry, Maria Curie-Skłodowska University in Lublin, Maria Curie-Skłodowska sq. 2, 20-031 Lublin, Poland; dariusz.osypiuk@mail.umcs.pl (D.O.); beata.cristovao@mail.umcs.pl (B.C.)

**Keywords:** coordination polymers, vanadium complexes, Schiff base *N*,*O*-donor ligands

## Abstract

This review provides an overview of the synthesis, characterization and application of coordination polymers based on *N*,*O*-donor Schiff base ligands. The coordination polymers (CPs) represent a novel class of inorganic–organic hybrid materials with tunable compositions and fascinating structures. They are composed of metal ions and organic ligands. Therefore, the nature of the metal ion and type of organic ligand is the most significant factor in constructing targeted coordination polymers with the desired properties. Due to the versatile coordination modes, *N*,*O*-donor Schiff base ligands are also used to construct various CPs.

## 1. Introduction

In recent decades, the design and construction of coordination polymers (CPs) has undergone significant development in the domain of crystal engineering. This advancement is not only attributed to their intriguing structural topologies but also to their potential applications in areas such as catalysis [1,2,3,4,5,6], luminescence [1,7], magnetism [1,4,8,9,10], gas adsorption and separation [4,11,12], biological activity [1,4,13,14,15,16] optics [4,17,18], sensors [4,19,20,21], nanomaterials [22], photoconduction [13,23], and photoelectrochemical materials [24,25]. CPs are composed of metal ions or cluster nodes, which are linked by organic ligands. Nonetheless, the synthesis of CPs with predictable structures and specific properties remains a significant challenge. This is due to the numerous subtle factors related to the crystallization process, including type of metal ions, type of organic ligands, type on solvents pH value, temperature, reaction time, and so on. The key to constructing a CP is to design suitable organic ligands, which could be, for example, Schiff bases, due to their versatile coordination modes. The aforementioned ligands are characterized by the possession of multiple coordination sites, a property that engenders high selectivity. This facilitates their capacity to react with a variety of metal ions, predominantly transition metals (e.g., vanadium, cadmium, zinc, lead, nickel, copper, manganese, mercury etc.), and lanthanides, thereby forming mononuclear, dinuclear, multinuclear, 1D chain, and 2D and 3D network structures [18,26,27,28,29,30,31,32]. A comprehensive review of the extant literature and the CSD database [33] revealed that *N*,*O*-donor Schiff bases predominantly form discrete complexes. Much less information is available on coordination polymers. In the coordination chemistry, vanadium mainly in the high valence state is attractive due to the use of numerous vanadium complexes as potential catalytic reagents in oxidation reactions of alcohols, olefins, benzene/alkyl aromatic compounds and sulphides [34,35,36,37,38,39,40,41,42,43,44,45]. This element can interconvert among various oxidation states and readily access higher oxidation states. Additionally, vanadium displays a range of coordination numbers, possesses a strong affinity for oxygen, and can act as a Lewis acid. These characteristics facilitate its application in redox and Lewis acid-catalyzed or -promoted reactions. Vanadium complexes, when combined with oxidants such as H_2_O_2_, serve as effective oxidation catalysts [46,47,48]. They readily generate intermediate species that feature an electron-rich peroxido group, which is capable of transferring oxygen to the organic substrate. Coordination polymers of vanadium are typically insoluble in common solvents. They possess the advantage over monomer analogues of easy separation from the catalytic reaction mixture, allowing for operational flexibility and recyclability. Vanadium complexes are also valuable models for understanding the biological functions of vanadium [49,50,51,52,53,54,55,56,57,58,59,60,61,62,63,64,65]. Scientists pay particular attention to the lower toxicity and harmless side effects of vanadium chelating complexes. In animals, vanadate inhibits various important metabolic enzyme systems in the liver or muscle, to utilize or accumulate glucose in the cells, while also blocking the effects of hormones that counteract insulin action [65]. Vanadium complexes with *N*,*O*-donor ligands of the Schiff base type mainly form discrete structures, while coordination polymers are much less common [33]. Furthermore, in the synthesis of vanadium coordination polymers, additional metal ions are frequently utilized, including Zn^2+^, Cd^2+^, and Na^+^, among others. This results in the formation of heteronuclear polymers. In contrast, ions of other metals, such as Mn^2+^, Ag^+^, Pb^2+^, Cd^2+^, and Cu^2+^, exhibit a greater propensity to yield homonuclear coordination polymers [33]. This review presents the synthesis, crystal structures, and potential applications of vanadium coordination polymers as well as examples of coordination polymers formed by other metal ions.

## 2. Synthesis of Coordination Polymers

**Figure 1:**
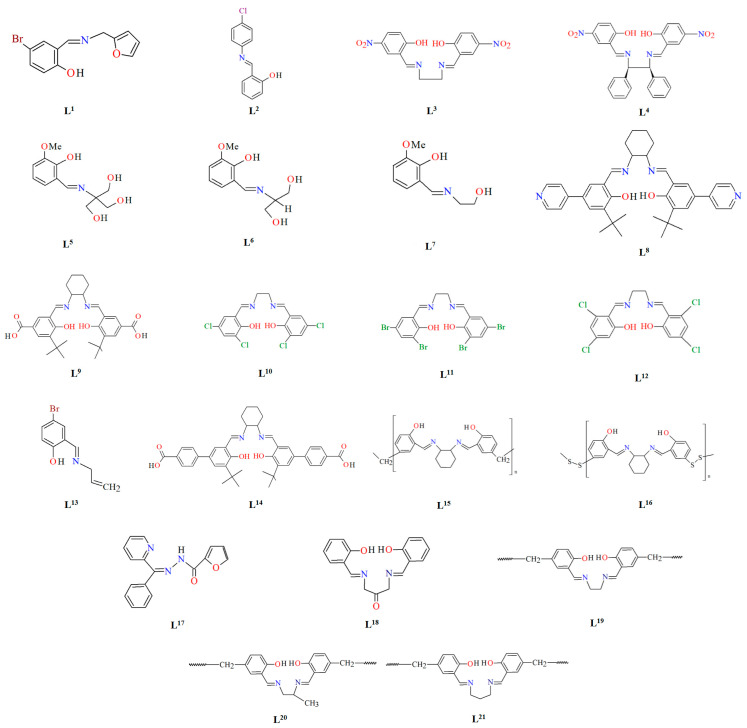
Scheme of *N*,*O*-donor Schiff base ligands using for coordination of vanadium ions **L^1^** [66], **L^2^** [67], **L^3^** [68], **L^4^** [68], **L^5^** [69], **L^6^** [69], **L^7^** [69], **L^8^** [70,71,72], **L^9^** [71,72], **L^10^** [73], **L^11^** [73], **L^12^** [73], **L^13^** [74], **L^14^** [75], **L^15^** [76], **L^16^** [76], **L^17^** [77], **L^18^** [78], **L^19^** [79], **L^20^** [79], **L^21^** [79].

**Figure 2:**
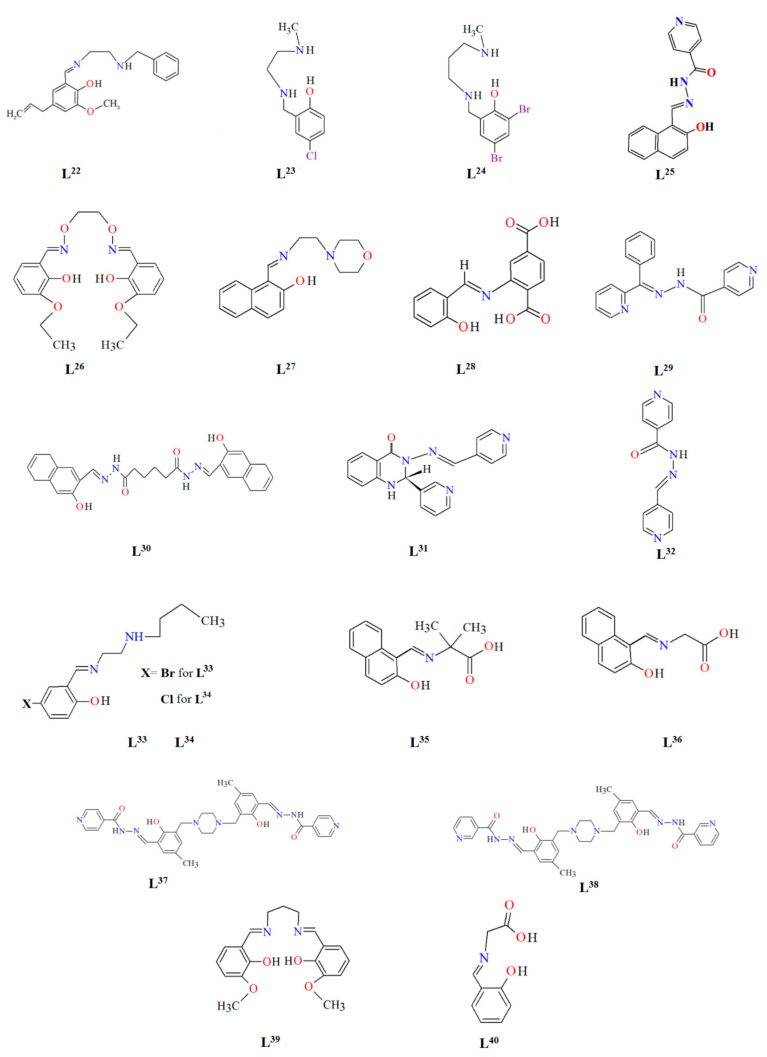
Scheme of *N*,*O*-donor Schiff base ligands using for coordination of selected metal ions **L^22^** [80], **L^23^** [81], **L^24^** [81], **L^25^** [1], **L^26^** [82], **L^27^** [83], **L^28^** [84], **L^29^** [85], **L^30^** [86], **L^31^** [87], **L^32^** [88], **L^33^** [4], **L^34^** [4], **L^35^** [89], **L^36^** [89], **L^37^** [90], **L^38^** [90], **L^39^** [91], **L^40^** [92].

Schiff bases, characterized by structural versatility, are of great interest to researchers due to their extensive complexation range and high stability under various oxidizing and reducing conditions. Among these, the most popular are probably the salen-type N_2_O_2_, N_2_O_4_ donor ligands prepared by 1:2 condensation of the respective diamine and salicylaldehyde or salicylaldehyde derivatives. The use of *N*-substituted diamines led to the obtainment of tridentate N_2_O donor Schiff bases. It is possible to reduce these ligands to form more flexible “reduced Schiff bases”. These ligands can be used in the synthesis of coordination polymers, which have interesting applications in materials science. Unfortunately, in most cases the reaction of such ligands with metal ions does not lead to the formation of coordination polymers, but only to the formation of discrete mono-, di- or polynuclear units. Therefore, additional atoms or donor groups capable of coordinating metal ions are often introduced into such structures, examples being the ligands **L^8^**, **L^9^**, and **L^14^** (Figure 1). For these salen-type ligands, substituents pyridine (**L^8^**), a carboxyl group (**L^9^**) and benzoic acid (**L^14^**) have been introduced into the aromatic ring, which can coordinate additional metal ions (Table 1). Another way to obtain coordination polymers of symmetric Schiff bases is the synthesis of polymeric ligands such as **L^15^**, **L^16^**, **L^19^**, **L^20^**, or **L^21^**. To obtain coordination polymers, in addition to modifying the Schiff base, additional bridging ligands, such as N_3_^−^, O^2−^, SCN^−^, CH_3_O^−^, C_2_H_5_O^−^, N(CN)_2_^−^, or carboxylate ions are introduced (Table 1 and Table 2). In the case of asymmetric ligands: **L^1^**, **L^2^**, **L^5^**, **L^6^**, **L^7^**, **L^13^**, **L^17^**, **L^22^**, **L^23^**, **L^24^**, **L^25^**, **L^27^**, **L^28^**, **L^29^**, **L^30^**, **L^31^**, **L^32^**, **L^33^**, **L^34^**, **L^35^**, **L^36^**, **L^40^** (Figure 1 and Figure 2), additional bridging ligands (e.g., complexes **1**, **2**, **6**, **16**, **25**–**28**, etc., Table 1 and Table 2) or additional donor atoms in the Schiff base structure (e.g., **29**, **33**, **34**, **35**, **37**, **38**, **39**, etc., Table 2) are often introduced.

Metal coordination polymers based on Schiff bases are mainly obtained by indirect reaction of metal ions with previously prepared *N*,*O*-donor ligands (e.g., **1**, **3**, **4**, **13**, **25**, **28**, **30**) or in a one-pot reaction (**6**, **7**, **5**, **16**, **44**, **45**). Compounds are obtained in different solvents, e.g., methanol, DMF, ethanol, or their mixtures (Table 1 and Table 2).

## 3. Crystal Structure and Properties of Vanadium Coordination Polymers

**Figure 3:**
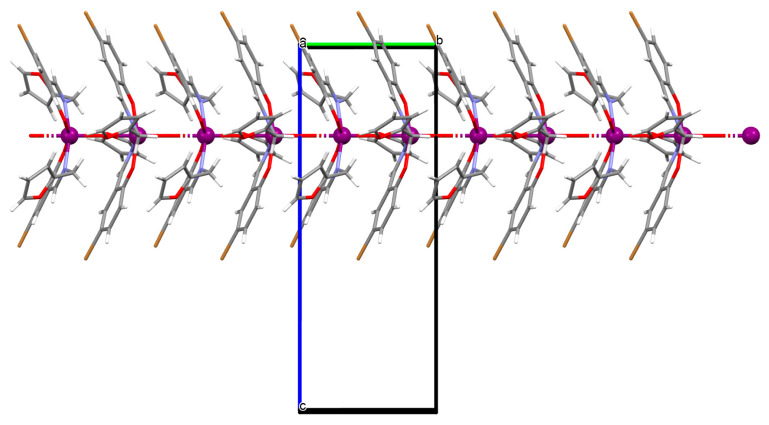
One-dimensional coordination polymer of **1** (Table 1) viewed along the *a*-axis [33,45,93].

1D coordination polymer [VOL^1^_2_]_n_ (**1**) was obtained during reaction of *N,O*-bidentate Schiff base (**L^1^** = 5-bromo-2-hydroxybenzyl-2-furyl(methyl)imine)) and VO^2+^ in methanol solution. **1** crystallizes in the monoclinic system. The complex is characterized by the presence of two symmetry-independent vanadium centres, which are located on a two-fold axis and have a distorted octahedral N_2_O_4_ coordination sphere. The oxygen atoms placed in the apical positions (the basal plane is formed by two nitrogen atoms and two oxygen atoms from two Schiff bases) link the metal centres to form an oxovanadium(IV) coordination polymer (Figure 3) [66].

The polynuclear oxovanadium(IV) Schiff base complex [VOL_2_]_n_ **1**, with TBHP as oxidant may be used as catalyst for epoxidation of cyclooctene [66].

**Figure 4:**
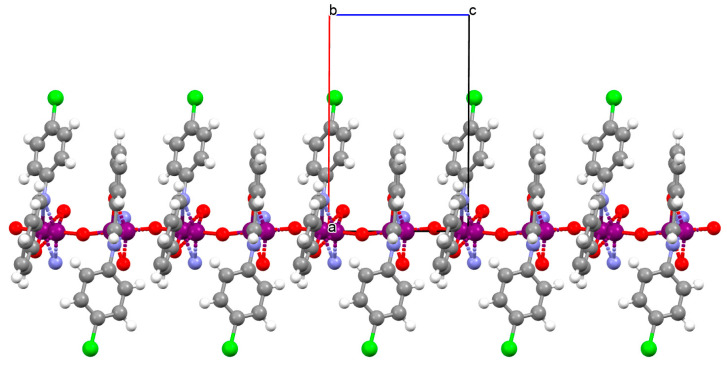
Fragment of crystal structure showing formation of a 1D chain of **2** (Table 1) viewed along the *b*-axis [33,67,93].

The oxygen-bridging coordination polymer {[(VO)_2_(L^2^)_4_]}_n_ (**2**) was obtained during reaction of VO^2+^ and *N*-(4-chlorophenyl)-salicylaldimine (**L^2^**) in the presence of Na[O_2_CMe] (ethanol as solvent was used). **2** crystalizes in the triclinic system, in the form of the needles (the monomeric compound crystalizes in the prism form). In crystal **2**, the monomeric units are connected through oxygen atoms V=O···V=O forming a chain along the *c*-axis (Figure 4). The magnetic measurements of the oxygen bridging indicated an occurrence of ferromagnetic interaction between metal centres. Conversely, no such interaction was observed in the monomeric compound [67].

**Figure 5:**
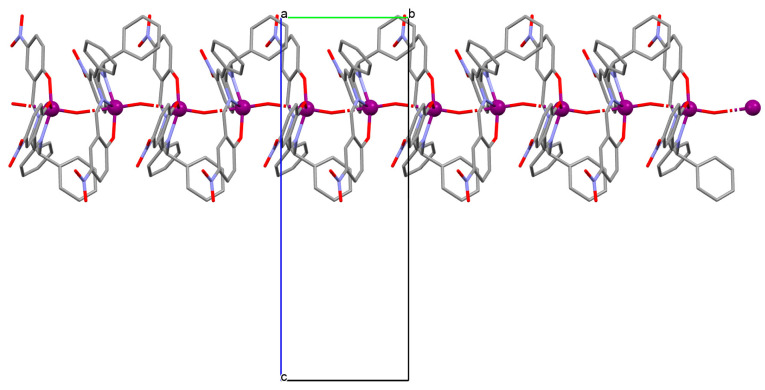
Fragment of crystal structure showing formation of a 1D chain of **4** (Table 1) viewed along the *b*-axis [33,68,93]. Solvent molecules are omitted for clarity.

[VO(L^3^)] 2H_2_O (**3**) and [VO(L^4^)] 0.5H_2_O (**4**) were obtained during reaction of tetradentate *N,N,O,O*-Schiff bases *N*,*N*′-di-5-nitrosalicylidene-1, 2-ethanediamine (H_2_(5-NO_2_salen = **L^3^**) or *N*,*N*′-di-5-nitrosalicylidene-(***R***,***S***)(***S***,***R***)-1, 2-diphenyl-1,2-ethanediamine (H_2_(5-NO_2_sal-*meso*-stien = **L^4^**) and VO^2+^ in methanol solution. **4** crystalizes in the monoclinic system. Complex **4** forms a polymeric structure with a distorted octahedral coordination geometry around the metal centres. Two azomethine nitrogens and the phenoxide oxygen of the Schiff base ligand and two oxo ions surround each vanadium(IV) ion. Oxo ions assemble V^IV^-Schiff base units into 1D chains running along to the *b*-axis (Figure 5). The results of the spectroscopic and magnetic studies indicate that the linear chain structures **3** and **4** are destroyed during the grinding process, resulting in the formation of different fragments of the polymeric chains. The mechanochemical reaction that occurred is hypothesized to be the cleavage of the ···V–O···V–O···bonds [68].

**Figure 6:**
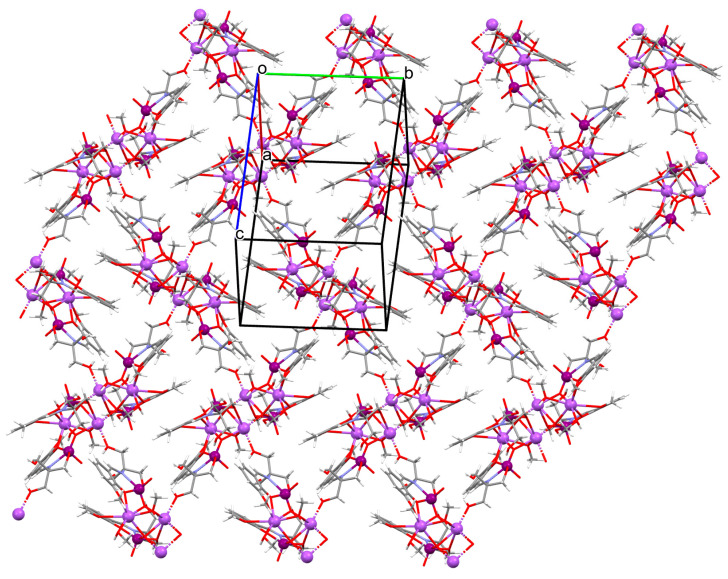
Fragment of crystal structure showing formation of a 2D network of **5** (Table 1) [33,69,93].

**Figure 7:**
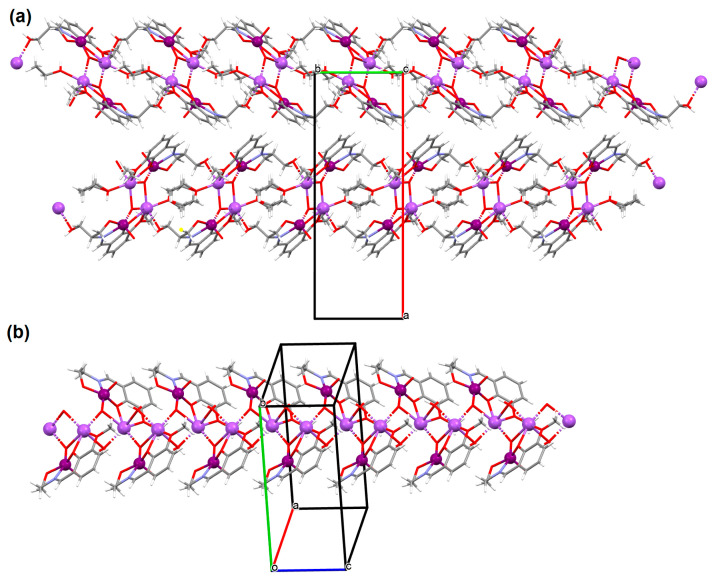
Fragment of crystal structure showing formation of 1D chain of (**a**) **6**, (**b**) **7** (Table 1) [33,69,93].

{[(VO_2_)_2_(L^5^)_2_]Na_2_(CH_3_OH)_2_}_n_ (**5**), {[(VO_2_)(L^6^)]Na(C_2_H_5_OH)}_n_ (**6**), and {[(VO_2_)(L^7^)(H_2_O)]Na}_n_ (**7**) were obtained during reaction of the respective Schiff bases (formed in situ from *o*-vanillin and tromethamine (**L^5^**), 2-amino-1,3-propanediol (**L^6^**), and ethanolamine (**L^7^**)) and VO^2+^ and sodium hydroxide in the methanol (**5**) or ethanol (**6**, **7**) solution. **5** crystallizes in the monoclinic system and its molecular structure is composed of [(VO_2_)_2_(L^5^)_2_]Na_2_(CH_3_OH)_2_ units, which form a 2D network (Figure 6). In **5** the coordination geometry around the two crystallographically independent vanadium centres is square pyramidal and consists of two doubly bonded oxido groups and one tridentate Schiff base ligand. The sodium ions are eight- and six-coordinated with triangular dodecahedral and distorted octahedral geometries, respectively. **6** crystallizes in the monoclinic system. As above, the coordination polyhedron around each vanadium ion can be described as a square pyramid, whereas the coordination polyhedron around each six-coordinated sodium ion adopts the geometry of a distorted trigonal prism. Schiff bases bind vanadium and sodium ions in infinite 1D chains (Figure 7a). **7** crystallizes in the orthorhombic system. In the crystal **7** the double deprotonated Schiff base acts as a tetradentate *N,O,O,O*-donor ligand and each vanadium ion has a square pyramidal geometry, while the coordination polyhedron around each sodium ion has the geometry of a distorted trigonal prism. A water molecule bridges adjacent sodium ions of {[(VO_2_)(L^7^)]Na(C_2_H_5_OH)} units to form a final 1D polymeric structure (Figure 7b). The obtained compounds may be potentially investigated as anti-diabetic vanadodrugs [69].

A homochiral vanadium–salen-based cadmium BPDC MOF (**8**) was obtained via in situ synthesis under solvothermal conditions during the reaction of the chiral salen ligand **L^8^** = (*R*,*R*)-(−)-1,2-cyclohexanediamino-*N*,*N*′-bis(3-tert-butyl-5-(4-pyridyl)salicylidene), VO^2+^, Cd^II^ and biphenyl-4,4′-dicarboxylic acid (BPDC) in the presence of DMF/EtOH/H_2_O. **8** crystallizes in the orthorhombic system. The asymmetric unit contains two Cd^II^ ions, two V–salen units (VOL), and two biphenyldicarboxylate (BPDC) ligands.

**Figure 8:**
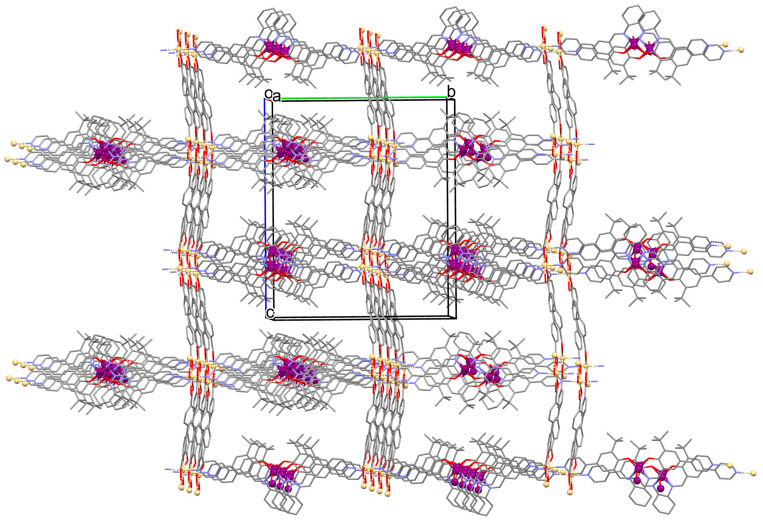
Part of crystal structure of 3D coordination polymer of **8** (Table 1) viewed along the *a*-axis [33,70,93]. Hydrogen atoms are omitted for clarity.

The vanadium(II) ion is bound by the O,N,N,O donor atoms of the Schiff base and the coordination sphere is completed by an oxygen atom. The cadmium(II) ions are surrounded by five oxygen atoms of three carboxylate groups and two pyridine nitrogen atoms of two Schiff bases, which result formation. The BPDC ligand bridges two cadmium ions and results in the formation of a 3D polymeric structure (Figure 8) [70].

The BET surface area of **8** is equal to 574 m^2^ g^−1^, and **1** has high H_2_ adsorption capacity (1.05 wt% at 77 K, 1 bar) and CO_2_ uptake (51 cm^3^ g^−1^ at 273 K, 1 bar), so it can potentially be used for the gas storage and gas separation. In addition, **8** may have a potential application in solvent-free cyanosilylation catalysis [70].

**Figure 9:**
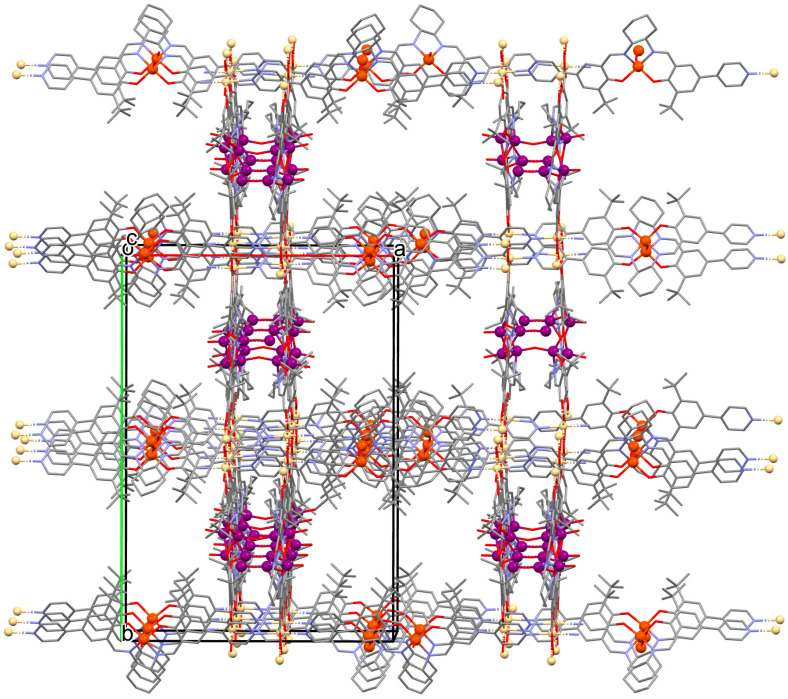
Part of crystal structure of 3D coordination polymer of **9** (Table 1) [33,71,93]. Solvent molecules and hydrogen atoms are omitted for clarity.

Two chiral porous metallosalen-based binary MOFs [Cd_2_(FeL^8^)_2_(VOL^9^)_2_](DMF)_2_(MeOH)_3_(H_2_O)_4_ (**9**) (Figure 9) and [Cd_2_(MnL^8^)_2_(VOL^9^)]·(DMF)(EtOH)_2_(H_2_O)_3_ (**10**) were obtained during reaction of Cd^II^, VO(H_2_L^9^) and FeL^8^(OAc) in DMF/MeOH or Cd^II^, VO(H_2_L^9^) and MnL^8^Cl in DMF/EtOH. **9** crystallizes in the orthorhombic system. The six-coordinated Cd^II^ centres **9** adopt a distorted octahedral coordination geometry, and their coordination sphere consists of four oxygen atoms derived from the carboxylate groups of three VOL^2^ units and two pyridyl nitrogen atoms from two FeL^8^ units. **10** crystallizes in the orthorhombic system. The coordination sphere of the seven-coordinated Cd^II^ centres **10** is formed by five oxygen atoms (three O atoms of carboxylate group of two VOL^2^ units, two O atoms of the chelating nitrate group) and two pyridyl nitrogen atoms of two MnL^1^ units. The geometry around each Cd^II^ centre can be described as a pentagonal bipyramid [71].

Compounds **9** and **10** as catalysts have been demonstrated to be highly effective in a range of reactions, including asymmetric sequential alkene epoxidation and epoxide ring-opening reactions. In these reactions, the enantioselectivity achieved has been reported to reach a level of up to 99%, making them catalysts of significant interest [71].

**Figure 10:**
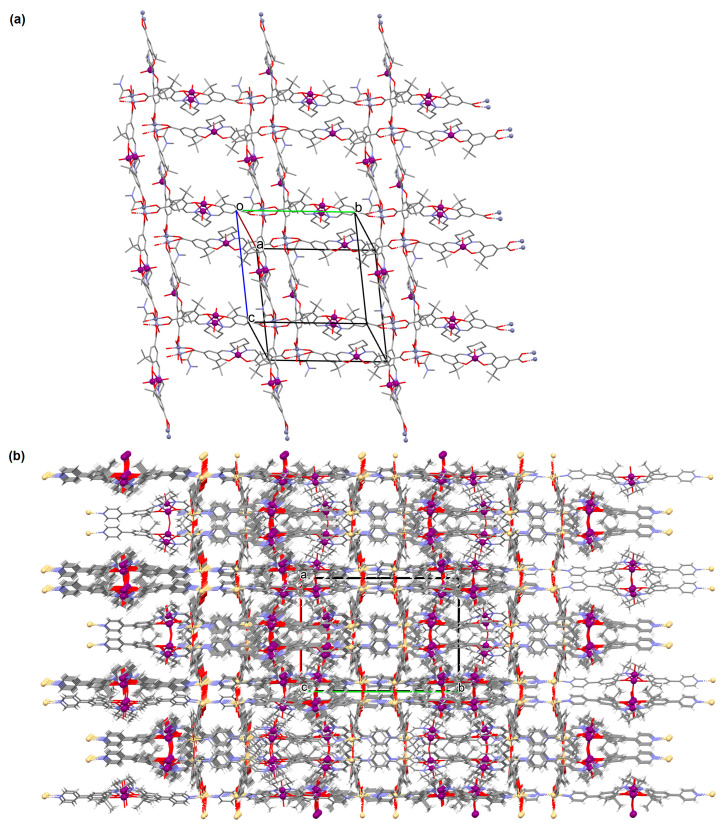
The coordination polymers: (**a**) 2D layered framework of **11**; (**b**) 3D framework of **12** (Table 1) [33,72,93]. Solvent molecules and hydrogen atoms are omitted for clarity.

[Zn_2_(VOL^9^)_2_]·DMA H_2_O (**11**) (where L^8^ = deprotonated salen dicarboxylate ligand) and [Cd_2_(VOL^8^)_2_(BPDC)_2_]·4DMF·2H_2_O (**12**) (where L^2^ = deprotonated salen dipyridine ligand) were obtained during reaction of [VO(H_2_L^9^)] and Zn^II^ or [VOL^8^], biphenyl-4,4′-dicarboxylic acid (H_2_BPDC), and Cd^II^ in the mixture of DMA/MeOH/H_2_O or DMF/MeOH, respectively. **11** crystallizes in the triclinic system. The five-coordinated V ion in the VOL^9^ core adopts a square-pyramidal geometry with the N,N,O,O atoms of the Schiff base ligand **L^9^** in the equatorial position and one double-bonded O atom in the apical position. In crystal **11**, the bidentate carboxylate groups of the {VOL^9^} cores connect Zn^II^ ions forming {Zn_2_(O_2_C)_4_} units that are next bridged by *exo*-tetradentate {VOL^9^} units resulting in the formation of a 2D layered framework (Figure 10a). Such 2D structures are arranged in space in such a way that they generate a 3D lamellar structure with open channels along the *a*-axis. Complex **12** crystallizes in the orthorhombic system. The polyhedral formed by donor atoms around the V centre in VOL^2^ unit may be also described as a square pyramid. In crystal **12**, six-coordinated Cd^II^ ions are linked by two bidentate carboxylate groups of two BPDC resulting in 2D layer structure. Adjacent 2D layers are bound into a 3D structure (Figure 10b) by cadmium ions linked by pyridyl nitrogen atoms of {VOL^8^} units. Following the oxidation of V^IV^ to V^V^, it was determined that the compounds may have been potentially used as highly effective, recyclable, and reusable as catalysts for the asymmetric cyanosilylation of aldehydes [72].

**Figure 11:**
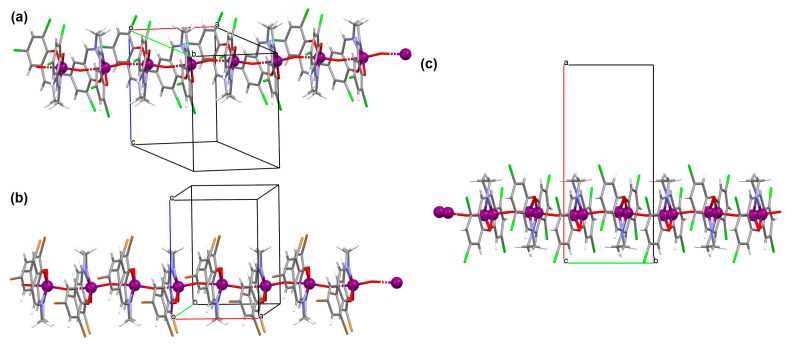
One-dimensional coordination polymers of: (**a**) **13**; (**b**) **14**; (**c**) **15** (Table 1) [33,73,93].

[VO(L^10^)] (**13**), [VO(L^11^)] (**14**), and [VO(L^12^)] (**15**) were obtained during reaction of 3,3′,5,5′-tetrachloro-,3,3′,5,5′-tetrabromo- and 4,4′,6,6′-tetrachlorosalen derivatives with VO^2+^ in methanol. **13** and **14** are isomorphous and crystallize in the orthorhombic system. The coordination environment around the vanadium(IV) centres consists of O,N,N,O do-nor atoms of the Schiff base ligand and two oxygen ions. The oxygen ions link the V^IV^ ions into chains running along the *a*-axis (Figure 11a,b). **15** crystallizes in the orthorhombic system. The coordination environment is similar to the above complexes, but the chains are formed along the [010] direction, and the vanadium ions are disordered with occupancy factors of 0.67/0.33 (Figure 11c). The presence of ferromagnetic exchange interaction between the V^IV^ centres was observed [73].

**Figure 12:**
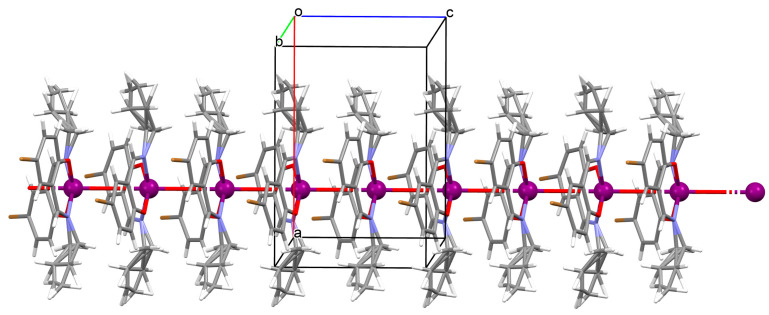
One-dimensional coordination polymers of **16** (Table 1) [33,74,93].

1D coordination polymer [VO(L^13^)_2_]_n_ (**16**) (where L^13^ = 5-bromo-2-((allylimino)methyl)phenolate)) was obtained in methanol in a one pot reaction using an allylamine, 5-bromo salicylaldehyde and VO^2+^. **16** crystallizes in the orthorhombic system. A distorted octahedral geometry is observed around the metal centre V^IV^ formed by *O,N,N,O*-donor atoms of Schiff base ligand and two oxygen ions. In crystal 1**6**, the metal ions are linked through the oxygen atoms, resulting in the formation of 1D chains running parallel to the *c*-axis (Figure 12) [74].

3D chiral porous framework [Cd_2_(VOL^14^)_2_]·5H_2_O (**17**) was synthesized during reaction of Cd^II^ and VO(H_2_L^14^) in a solution of DMF. **17** crystallizes in the orthorhombic system. In **17**, the fundamental structural element is constituted by infinite Cd-O-C chains that are connected by [VOL^14^] units. The coordination environment surrounding each vanadium ion is characterized by a square-pyramid geometry, with the equatorial plane occupied by the N,N,O,O atoms of a Schiff base and the apical position occupied by one double bond oxygen atom. In contrast, the coordination environment surrounding a cadmium ion adopts a distorted octahedral geometry. The oxidation of V^IV^ to V^V^ VO-MOF showed enhanced stereoselectivity and comparable activity in the cyanation of aldehydes. **17** can be easily recycled and reused without significant loss of catalytic activity and enantioselectivity [75].

In the case of polymeric Schiffa bases **L^15^**, **L^16^**, **L^19^**, **L^20^**, or **L^21^** [76,79], the coordination of metal ions has not resulted in monocrystals, and there are no structures of such coordination polymers. However, these compounds (**18**, **19**, **22**–**24**) show interesting catalytic properties that may be of application, as is often observed for other vanadium coordination polymers (**20**, **21**) [77,78].

**Figure 13:**
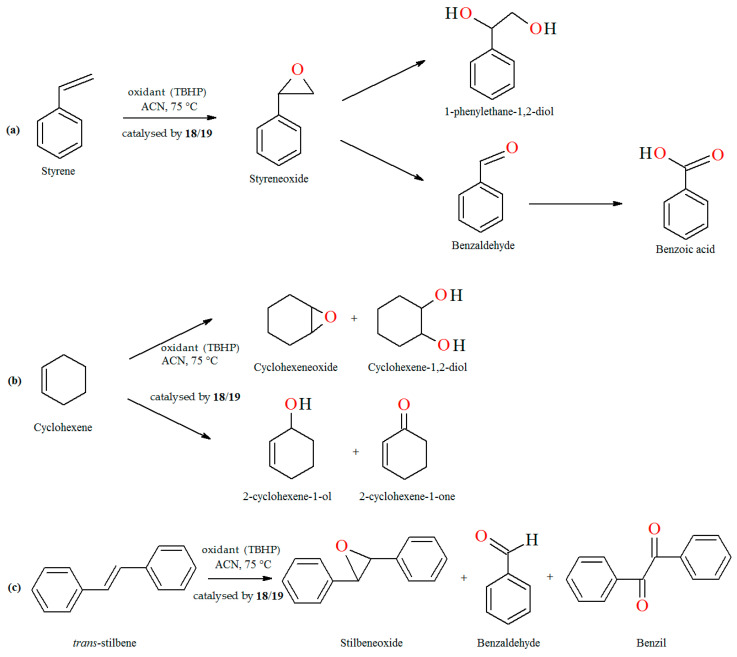
The oxidation reaction of (**a**) styrene; (**b**) cyclohexene; (**c**) *trans*-stilbene catalyzed by **18** and **19**. Adapted from reference [76].

The oxidation of styrene with *tert*-butylhydroperoxide (TBHP) as oxidant catalyzed by coordination polymers [–CH_2_{VO(sal-dach)·DMF}–]_n_ (**18**) and [–S_2_{VO(sal-dach)·DMF}–]_n_ (**19**) results in formation of styrene oxide, 1-phenylethane-1,2-diol, benzaldehyde, benzoic acid along with some unidentified products. Reaction conditions: styrene (10 mmol); catalyst (equivalent to 0.032 mmol of repeating unit); acetonitrile (20 mL); temperature (75 °C) (Figure 13a). The oxidation of cyclohexene catalyzed by [–CH_2_{VO(sal-dach)·DMF}–]_n_ (**18**) and [–S_2_{VO(sal-dach)·DMF}–]_n_ (**19**) afforded various oxidation products, i.e., cyclohexeneoxide, 2-cyclohexene-1-ol,cyclohexane-1,2-diol, and 2-cyclohexene-1-one. Reaction conditions: cyclohexene (10 mmol); catalyst (0.032 mmol of repeating unit); acetonitrile (20 mL); temperature (75 °C) (Figure 13b). The oxidation of *trans*-stilbene TBHP as oxidant catalyzed by [–CH_2_{VO(sal-dach)·DMF}–]*_n_* (**18**) and [–S_2_{VO(sal-dach)·DMF}–]*_n_* (**19**) gives mainly stilbeneoxide, benzaldehyde, and 1,2-diphenylethanedione (benzil) as oxidation products (Figure 13c). Reaction conditions: *trans*-stilbene (5 mmol); catalyst (0.032 mmol of repeating unit); acetonitrile (20 mL); temperature (75 °C) [76].

**Figure 14:**
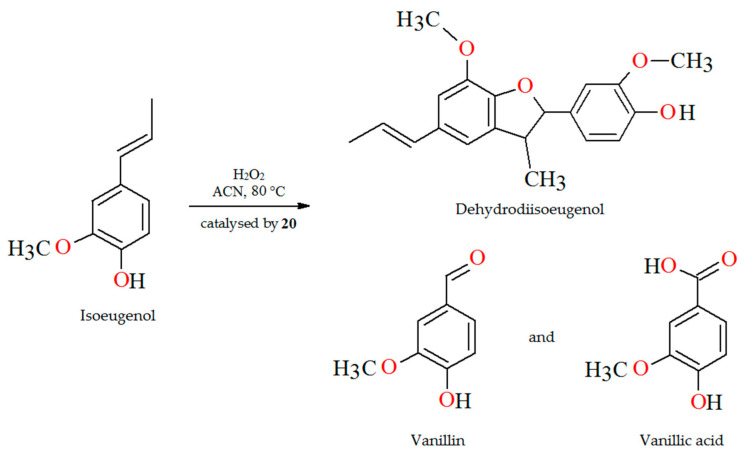
Scheme of the oxidation of isoeugenol catalyzed by **20**. Adapted from reference [77].

The oxidation of isoeugenol in the presence of aqueous H_2_O_2_ as oxidant (Figure 14), catalyzed by polymer-supported complex PS-im[V^V^O_2_(L^17^)] (**20**) gave dehydrodiisoeugenol, vanillic acid, and vanillin. Reaction conditions: isoeugenol (5 mmol); 30% H_2_O_2_ (10 mmol); catalyst PS-im[V^V^O_2_(L^17^)] (0.010–0.030); acetonitrile (7 mL); temperature (80 °C) [77].

**Figure 15:**
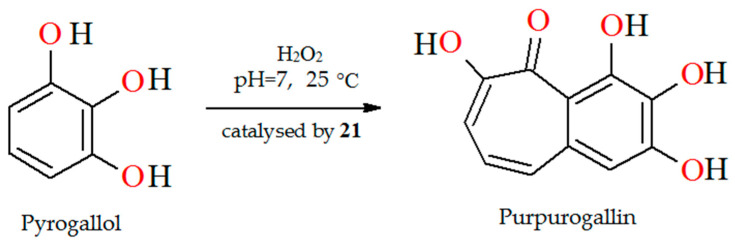
Peroxidase like oxidation of pyrogallol to purpurogallin catalyzed by **21**. Adapted from reference [78].

The oxidation of pyrogallol with H_2_O_2_ as oxidant catalyzed by polymer-supported complex PS-[V^IV^O(L^18^)] (**21**) results in the formation of purpurogallin (Figure 15). Reaction conditions: pyrogallol (25 mmol) in phosphate buffer (pH 7), 30% H_2_O_2_ (25 mmol), catalyst PS-[V^IV^O(L^18^)] (0.005–0.020 g), temperature (25 °C) [78].

**Figure 16:**
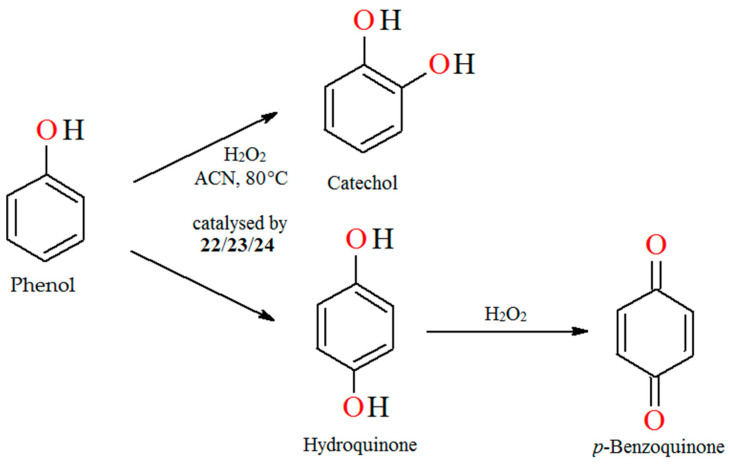
Oxidation of phenol catalyzed by **22**–**24**. Adapted from reference [79].

The liquid-phase catalytic hydroxylation of phenol with H_2_O_2_ as oxidant catalyzed by oxovanadium(IV) polymeric complexes [–CH_2_{VO(salen)}–]_n_ (**22**), [–CH_2_{VO(sal-1,2-pn)}–]_n_ (**23**) and [–CH_2_{VO(sal-1,3-pn)}–]_n_ (**24**), respectively, leads to catechol and hydroquinone as products of oxidation (Figure 16). Reaction conditions: phenol (0.05 mol); 30% aqueous H_2_O_2_ (0.05 mol); acetonitrile (2 mL); catalyst **22**, **23,** and **24** (0.01 g); temperature (80 °C) [79].

## 4. Crystal Structure and Properties of Selected Metal Ion Coordination Polymers

**Figure 17:**
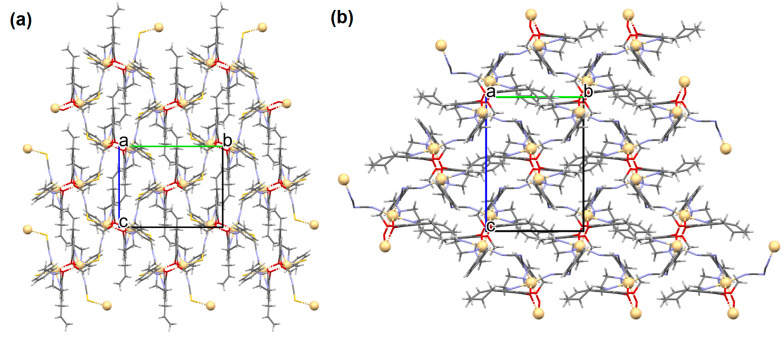
Fragment of crystal structure of cadmium(II) 2D coordination polymers: (**a**) **25**; (**b**) **26** (Table 2) [33,80,93].

[Cd_2_(L^22^)_2_(SCN)_2_]_n_ (**25**) and [Cd_2_(L^22^)_2_(N(CN)_2_)_2_]_n_ (**26**) were prepared at ambient temperature by direct reaction of methanolic solution of Cd^II^ and 4-allyl-2-(((2-(benzylamino)ethyl)imino)methyl)-6-methoxyphenol **L^22^** = C_20_H_24_N_2_O_2_ in the presence of SCN^−^/N(CN)_2_^−^ (stoichiometric ratio 1:1:1). **25** and **26** crystallize in the monoclinic system. The distorted octahedral geometry around the metal centre Cd^II^ is formed by two bridged phenoxido oxygens, an imine and an amine nitrogen of the deprotonated Schiff base **L^22^**, and by nitrogen and sulphur atoms of two *μ*_1,3_ bridged thiocyanate ions **25**/nitrogen atoms of two *μ*_1,5_ bridged dicyanamide ions **26**. In the crystal structure of the coordination polymers **25** and **26** bridged thiocyanate/dicyanamide ions link neighbouring units [Cd_2_(L)_2_]^2+^ to form 1D chains. In addition, the units of each layer are linked together again by another *μ*_1,3_-bridged thiocyanate **25** *μ*_1,5_-bridged dicyanamide **26** ions, respectively, which creates 2D architecture (Figure 17) [80].

[Cd_2_(L^22^)_2_(SCN)_2_]_n_ **25** and [Cd_2_(L^22^)_2_(N(CN)_2_)_2_]_n_ **26** may be used for monitoring toxic 2,4,6-trinitrophenol (TNP) in low detection threshold [80].

**Figure 18:**
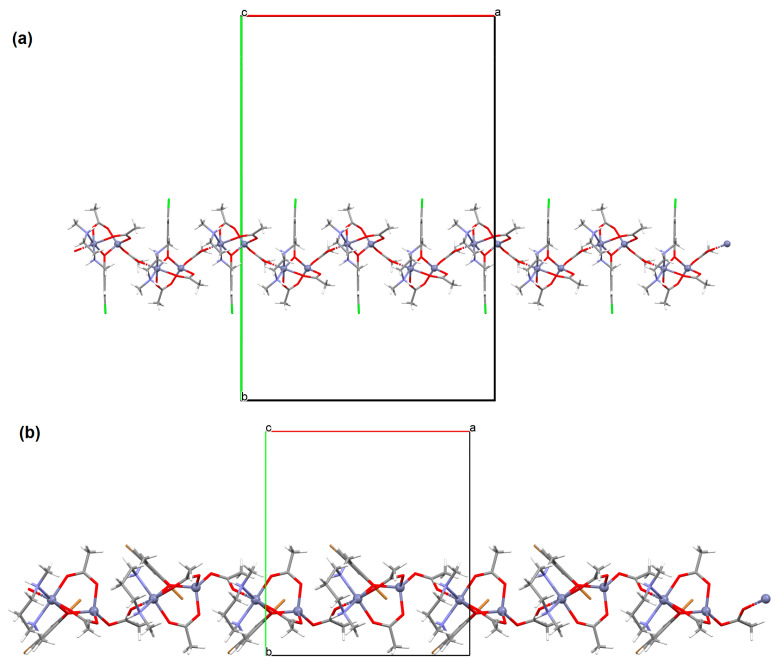
(**a**) Part of crystal structure of **27** (Table 2) showing the formation of chain along [100] direction. Solvent molecules are omitted for clarity; (**b**) The 1D chain of **28** (Table 2) viewed along the *c*-axis [33,81,93].

[Zn_2_L^23^(*μ*-OAc)_3_]_n_·H_2_O (**27**) and [Zn_2_L^24^(*μ*-OAc)_3_]_n_ (**28**) forming 1D chains (Figure 18) were synthesized during reaction of Zn^II^ and tridentate N,N,O reduced Schiff base ligands (stoichiometric ratio 2:1) **L^23^** {4-chloro-2-(((2-(methylamino)ethyl)amino)methyl)phenol} and **L^24^** {2,4-dibromo-6-(((3-(methylamino)propyl)amino)methyl)phenol}, respectively, in methanol. **27** and **28** crystalize in orthorhombic system. In both compounds, the coordination number of zinc ions is equal to 6 and 4, respectively, with the distorted octahedral and the distorted tetrahedral geometry around the metal centres. The six-coordinated Zn^II^ is equatorially coordinated by two amine nitrogen, one bridging phenoxo oxygen atom of an *N,N,O*-donor ligand, and one oxygen atom of bridging acetate ion, and axially it is coordinated by two oxygen atoms of another bridging acetate. The four-coordinated Zn^II^ is surrounded by one phenoxo oxygen atom of *N,N,O*-donor ligand and three oxygen atoms of different bridging acetate ions. The synthesized coordination polymers could potentially be investigated as compounds used in the photocatalytic degradation of various organic dyes, in the bio-mimetic catalysis, for producing a variety of optoelectronic devices and as a sensor for the detection of various nitro-aromatic explosives [81].

**Figure 19:**
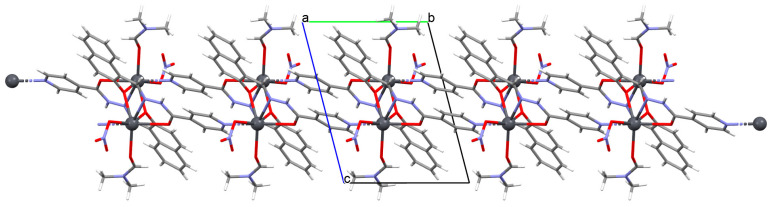
The 1D looped chain of **29** (Table 2) viewed along the *a*-axis [1,33,93].

{[Pb(L^25^)NO_3_DMF]}_n_ (**29**) (where **L^25^** = anion of isonicotinic acid (2-hydroxy-naphthalen-1-ylmethylene)-hydrazide) was obtained during reaction of Pb^II^ and *N,N,O,O*-donor Schiff base (isonicotinic acid (L^25^) dissolved in the mixture MeOH/DMF. **29** crystalizes in the triclinic system. A seven-coordinated Pb^II^ is surrounded by five oxygen and two nitrogen atoms that form a distorted pentagonal bipyramidal geometry around the metal centre. Each Schiff base coordinates three metal ions leading the formation of a 1D looped chain running along [010] (Figure 19). Compound **29** indicates the n-type semiconducting behaviour, as well as the stability against photo corrosion, so it may be potentially considered as the next generation of energy materials [1].

**Figure 20:**
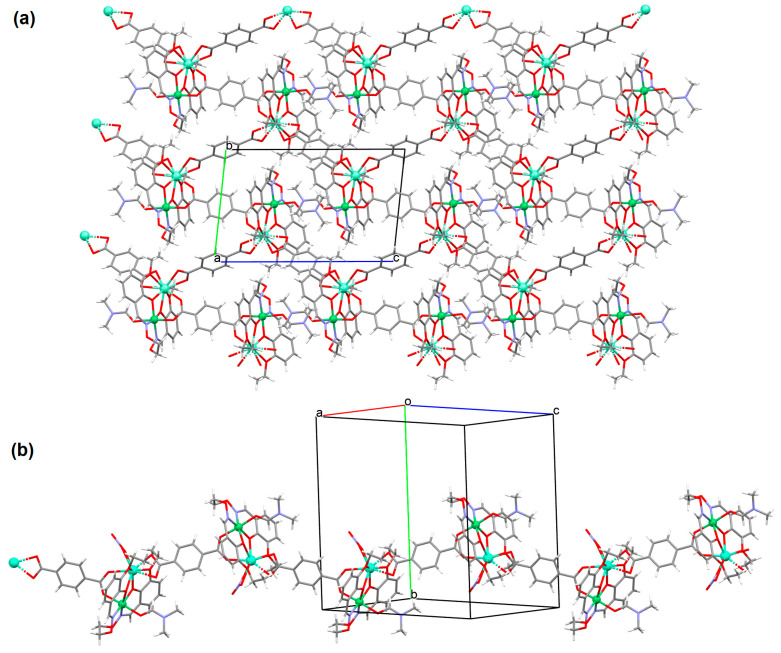
Part of crystal structure of coordination polymers: (**a**) showing formation of 2D coordination polymer of **30** (Table 2); (**b**) The 1D chain of **31** (Table 2) [33,82,93].

The heteronuclear coordination polymers ^2^_∞_[Ni(L^26^)Sm(bdc)_1.5_]CH_3_OH (**30**) and ∞[Ni(L^26^)Tb(NO_3_)(DMF)(bdc)] (**31**) (where bdc = anion of terephthalic acid, **L^26^** = anion of 3-EtO salamo) were synthesized during reaction of methanolic **30**/ethanolic **31** solution of Ni^II^ and Sm^III^/Tb^III^ salts with salamo-like bisoxime (dissolved in trichloromethane) and terephthalic acid H_2_bdc (dissolved in DMF). **30** crystallizes in the triclinic system whereas **31** crystallizes the monoclinic system. In crystals **30** and **31** [Ni^II^(L^26^)Sm^III^]/[Ni(L^26^)Tb(NO_3_)] units are connected by exodentate (bdc)^2−^ ions forming 2D **30** and 1D **31** chain coordination polymers (Figure 20). The fluorescence intensities of **30** and **31** are lower than those of the free salamo-like bisoxime **L^26^**, indicating that Ni^II^/Ln^III^ ions influence the changes in the fluorescence properties of the ligand [82].

**Figure 21:**
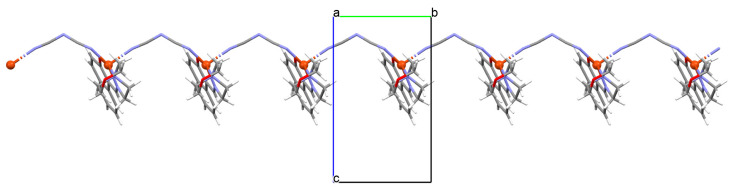
Structure of **32** (Table 2) showing formation of 1D coordination polymer viewed along the *a*-axis [33,83,93].

[Cu(L^27^)(*μ*_1,5_-dca)]_n_ an end-to-end dicyanamide bridged (dca) coordination polymer **32** (Figure 21) was obtained during reaction of a naphthalene-based *N,N,O*-Schiff base blocking ligand and Cu^II^ ions. **32** crystallizes in the monoclinic system in which a five-coordinated Cu^II^ is coordinated equatorially by two nitrogen and one oxygen atoms of the tridentate Schiff base and one nitrogen atom of the dicyanamide ion and the apical position is occupied by one nitrogen atom of another bridged dicyanamide ion. The coordination polyhedron has classical distorted square pyramidal geometry [83].

**Figure 22:**
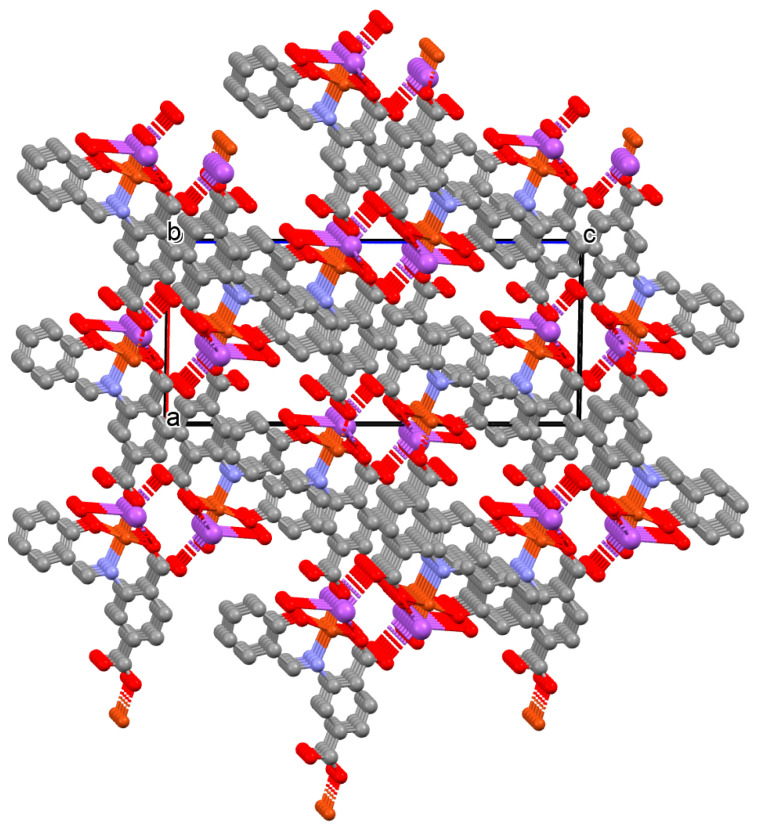
Three-dimensional coordination polymer of **33** (Table 2) viewed along the *b*-axis [33,84,93]. Hydrogen atoms are omitted for clarity.

3D heterometallic coordination polymer [CuNa(L^28^)(H_2_O)(OH)]_n_ (**33**) was obtained by using the tridentate *N,O,O*-donor Schiff base (2-[(*E*)-(2-hydroxyphenyl)methyleneamino]terephthalic acid), Cu^II^ and Na^I^ ions. **33** crystallizes in the monoclinic system. The penta-coordinated Cu^II^ ion adopts a distorted square pyramidal geometry with one nitrogen and two oxygen atoms of the Schiff base and one oxygen atom of another *N,O,O*-donor ligand in the equatorial position and one oxygen atom of the hydroxide group in the apical position. The four-coordinated Na^I^ ion adopts a tetrahedral geometry with two carboxylate oxygen atoms, one hydroxy oxygen and one water oxygen atom. In the crystal Cu^II^ and Na^I^ centres are connected by a bridging hydroxide group. Each Schiff base ligand adopts a tetradental coordination mode binding two copper(II) and two sodium ions. The presence of additional ligands bridging the metal ions, i.e., water molecules and hydroxyl ions, results in the formation of a 3D network (Figure 22). The antibacterial efficacy of **33** was studied in relation to both Gram-positive and Gram-negative strains in order to check if **33** can be potential used as drug and/or antimicrobial agent [84].

**Figure 23:**
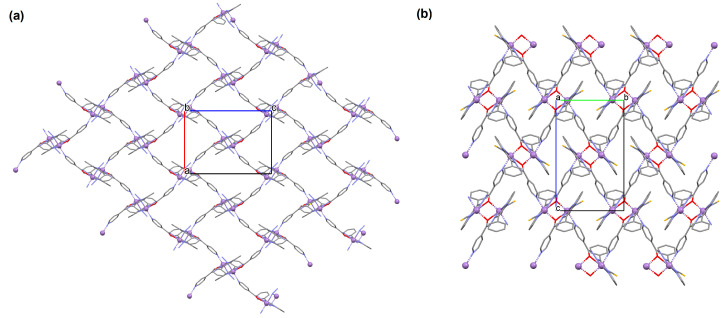
Part of crystal structure of 2D coordination polymers: (**a**) **34** (Table 2) viewed along the *b*-axis; (**b**) **35** (Table 2) viewed along the *a*-axis [33,85,93]. Hydrogen atoms are omitted for clarity.

[Mn_2_(L^29^)_2_(*μ*-N_3_)_2_]_n_ (**34**) 2D coordination polymer (Figure 23a) with azide bridged dinuclear unit was synthesized during reaction of the isonicotinhydrazide ligand (**L^29^**) with Mn^II^ and sodium azide in methanol. During the synthesis of [Mn_2_(L^29^)_2_(NCS)_2_]_n_ (**35**), a 2D coordination polymer (Figure 23b), instead of sodium azide, potassium thiocyanate was used. **34** crystalizes in the orthorhombic system whereas **35** crystalizes in the monoclinic system. A ditopic isonicotinhydrazone-based tetradentate *N,N,O*-donor ligand coordinates to the Mn^II^ centre in the enolic form (=N–N=C–O). The magnetic behaviours exhibited by the dimeric samples demonstrate a pronounced intradimer exchange of antiferromagnetic nature for **34**, while **35** exhibits a ferromagnetic coupling between metal centres [85].

**Figure 24:**
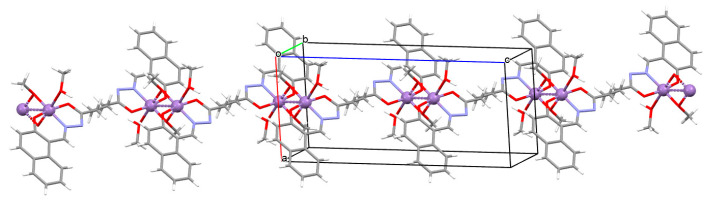
Part of crystal structure showing formation of 1D coordination polymer of **36** (Table 2) [33,86,93].

[Mn_2_(L^30^)(*μ*-OCH_3_)_2_(OHCH_3_)_2_]_n_ (**36**) 1D coordination polymer was obtained from the hexadentate dihydrazoneSchiff base (**L^30^**, bis[(2-hydroxynaphthalen-1-yl)methylene]-adipohydrazide) and Mn^II^ in methanol solution. **36** is a rare example of the Mn^III^ coordination polymer (during synthesis the Mn^II^ is oxidized to Mn^III^ by air) with methoxy-bridged groups. The coordination polyhedron built by the Mn^III^ centres exhibits Jahn–Teller distorted octahedral arrangement. Each Schiff base binds two manganese(II) ions and acts as a hexadentate ligand coordinating a single metal centre via O_phenoxide_, N_azomethine_, O_carbonyl_-donor atoms. The same metal centres are also bridged by two oxygen atoms of methoxide ions. This gives rise to Mn_2_(*μ*-O)_2_ metallocycles. This coordination of the manganese(II) ions by the Schiff base and the methoxide ion leads to the formation of chains that run along the *c*-axis (Figure 24). The magnetic measurements evident the presence of a weak antiferromagnetic interaction between Mn^III^ centres. It was also demonstrated that **36** exhibited catalase-like activity in the disproportionation of H_2_O_2_ [86].

**Figure 25:**
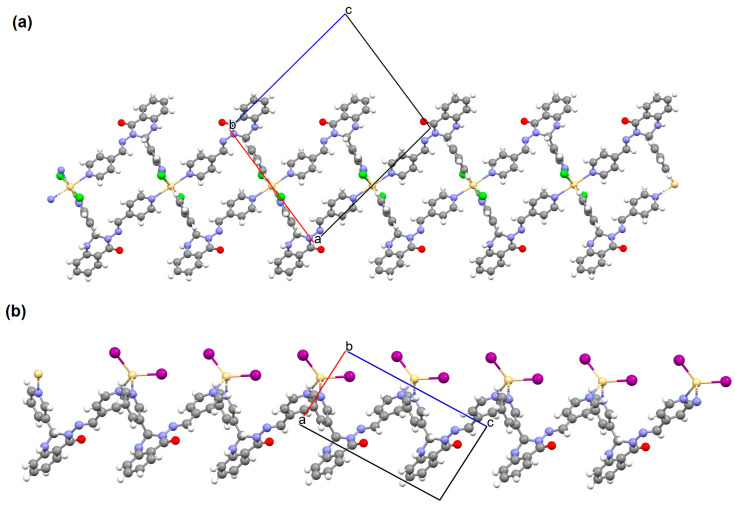
Fragment of crystal structure of cadmium(II) coordination polymers: (**a**) 1D looped chain structure of **37** (Table 2) viewed along the *b*-axis (solvent molecules are omitted for clarity); (**b**) 1D zig-zig chain structure of **38** (Table 2) viewed along the *b*-axis [33,87,93].

**Figure 26:**
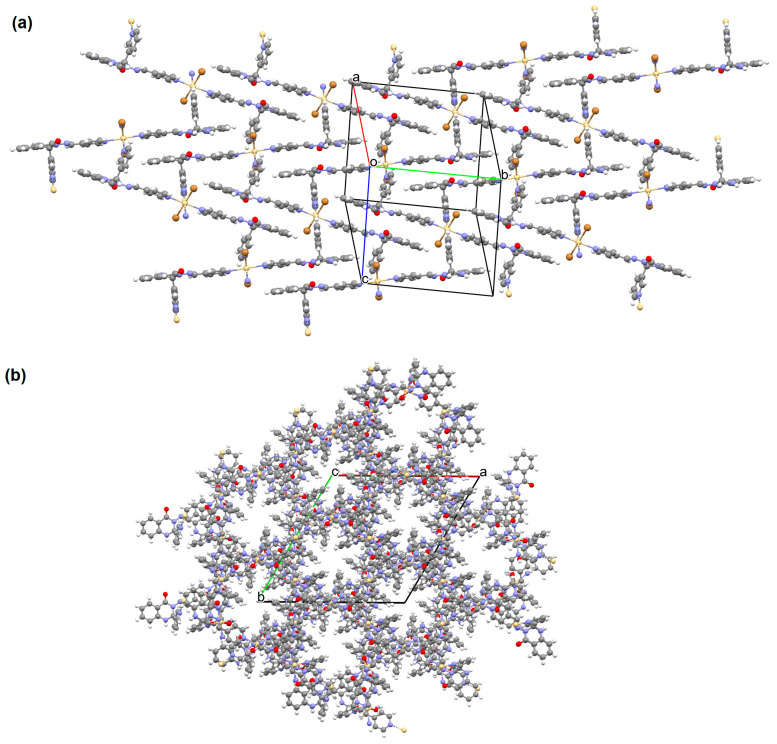
Fragment of crystal structure of cadmium(II) coordination polymers: (**a**) 2D structure of **39** (Table 2); (**b**) 3D network structure of **40** (Table 2) viewed along the *c*-axis [33,87,93]. Solvent molecules are omitted for clarity.

Lin et al. used a racemic bispyridyl ligand (**L^31^**) to synthesize four coordination polymers of cadmium(II). To synthesize the complexes, they used four different cadmium(II) salts (chloride, iodide, bromide, and nitrate) and demonstrated the influence of the counterion on the synthesis and structure of the obtained coordination polymers {[Cd(L^31^)_2_Cl_2_]·2DMF}_n_ (**37**), [Cd(L^31^)I_2_]_n_ (**38**), {[Cd(L^31^)_2_Br_2_]·4H_2_O}_n_ (**39**), and {[Cd(L^31^)_2_(H_2_O)_2_](NO_3_)_2_·2CH_3_OH·8H_2_O}_n_ (**40**). All compounds crystalized in the monoclinic system in different space groups. In complex **37**, the metal ions are coordinated by four nitrogens of different Schiff bases and two chloride ions, which formed an octahedral environment around Cd^II^. Adjacent cadmium(II) ions are linked by two ligand molecules to form 24-membered metallamacrocycles arranged in a chain along [101] direction (Figure 25a). The change of the counterion from chloride to iodide (complex **38**) also resulted in a 1D-corridinated polymer, but of a different type, i.e., zig-zag chains (Figure 25b). The coordination polyhedron around the Cd^II^ ion takes the form of a distorted tetrahedron and consists of by two pyridyl nitrogen atoms from different Schiff bases and two iodide ions. In the case of compound **39**, metal ions are surrounded via four Schiff base ligands and two water molecules. As for **37**, the coordination environment around the Cd^II^ ions is a slightly distorted octahedron, but the way the metal ions are bound and arranged results in two-dimensional coordination polymers (Figure 26a). In the last coordination polymer (**40**), the coordination environment around the central ion is also a distorted octahedron. The Cd^II^ ions are coordinated by the pyridyl nitrogen of the four Schiff bases and two water molecules. This coordination mode of the ligands with cadmium(II) ions results in the formation of a 3D framework (Figure 26b). The synthesized polymers were tested for sorption for iodine and dyes. The study showed that these compounds can be used to remove iodine and dyes from wastewater, e.g., complex **38** displayed selective adsorption for crystal violet and, complex **40** showed preferential adsorption for direct yellow in the mixed solution of these two dyes [87].

**Figure 27:**
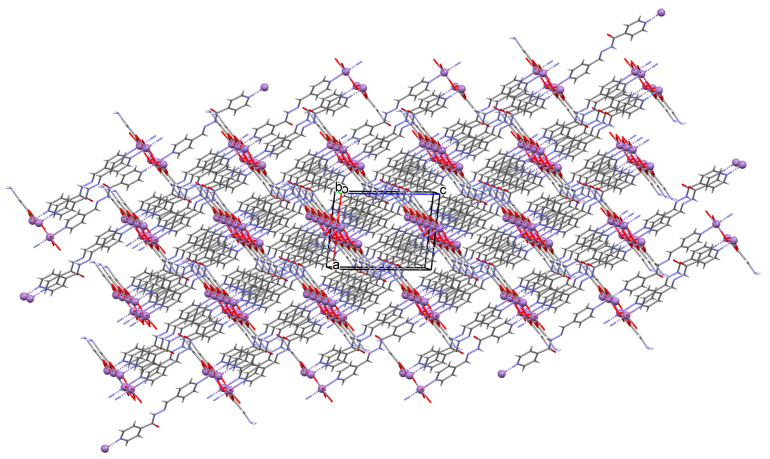
Fragment of crystal structure of **41** (Table 2) viewed along the *b*-axis [33,88,93].

Shit and co-workers reported the synthesis of Mn^II^ 3D coordination polymer with pyridine-4-carboxaldehyde isonicotinoyl hydrazine (**L^32^**) and 5-aminoisophthalic acid (H_2_aisp), [Mn_2_(aisp)_3_(L^32^)_2_(solvent)]_n_ (**41**). The compound crystallizes in triclinic system. The distorted octahedral environment observed around manganese(II) ions is formed by three carboxylate ions and two pyridyl nitrogens of two Schiff bases. The asip^2−^ ligand bridges two metal ions into a 2D framework. The **L^32^** also acts as a bridging ligand, which results in the formation of a 3D network present in Figure 27 [88].

**41** shows significant cytotoxicity against some cancer cell lines (PC3, HeLa, MDA-MB-231, and A549). The complex is particularly effective in HeLa cell death through induced apoptosis. The compound was also tested for use as a functional matrix for the encapsulation of the drug, i.e., diclofenac sodium. Studies have shown that at low pH, only a small amount of drug is released (less than 10%), while at pH 7.4 (as observed in the intestinal environment), a significant increase in drug release was observed, reaching approximately 87% over 30 h. These results show the promising potential of **41** as an oral drug carrier. Moreover, this coordination polymer displays intriguing electrical and optical properties, which can be utilized in the fabrication of energy-saving electronic devices [88].

**Figure 28:**
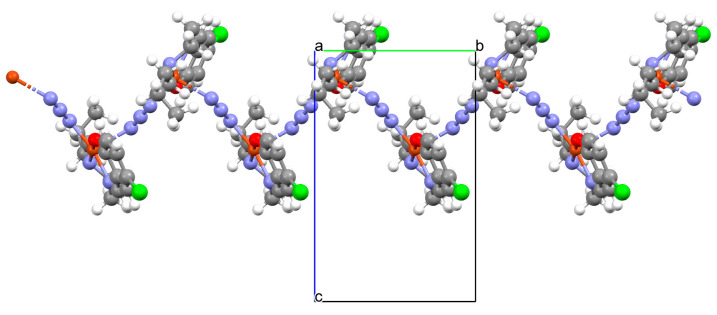
Fragment of crystal structure showing formation of a zig-zag chain of **43** (Table 2) viewed along the *a*-axis [4,33,93].

Other examples of metal-assembled Schiff base coordination polymers include a two isostructural copper complexes **42** and **43** with Schiff bases derived from *N*-butylethyldiamine. The reaction of copper(II) acetate with 4-bromo-2-(((2-(butylamino)ethyl)imino)methyl)phenol (**L^33^**)/4-chloro-2-(((2-(butylamino)ethyl)imino)methyl)phenol (**L^34^**) in the presence of NaN_3_ in methanol medium leads to the formation of zig-zag one-dimensional (1D) chains (Figure 28). In both complexes, copper(II) ions are surrounded by N,N,O atoms of a Schiff base molecule and two nitrogen atoms of azide ions, leading to the formation of a distorted square pyramidal coordination geometry around metal centres. The complexes show moderate catecholase-like activity as well as interesting optoelectronic properties [4].

**Figure 29:**
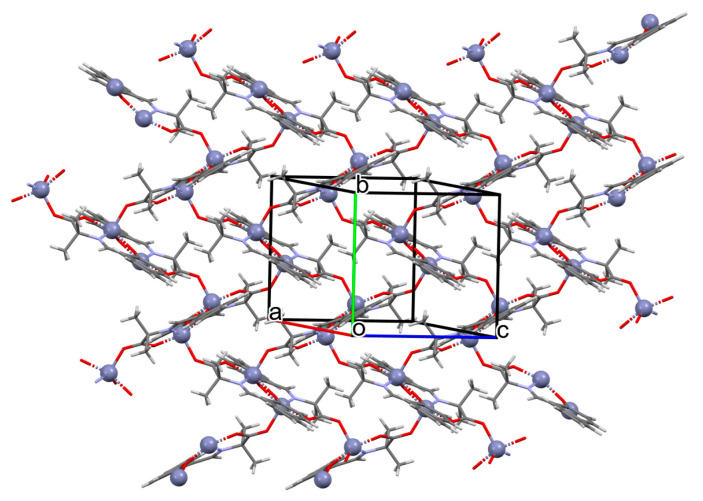
Fragment of crystal structure showing a 2D network of **44** (Table 2) [33,89,93].

**Figure 30:**
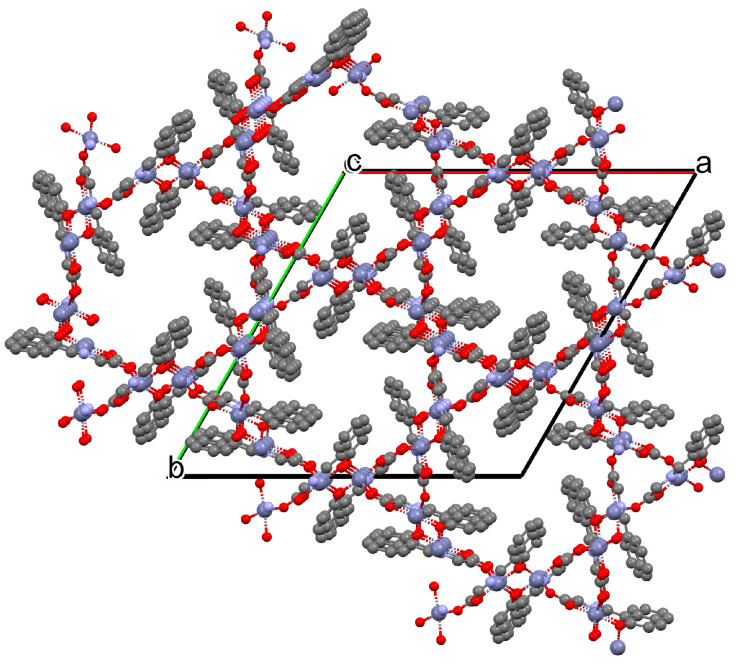
Fragment of crystal structure showing a 3D framework of **45** (Table 2) viewed along the *c*-axis [33,89,93]. Hydrogen atoms and solvent molecules are omitted.

Karakousi et al. reported the formation of 2D and 3D zinc coordination polymers that emit blue light. The catena-((*μ-N*-[(2-oxynaphthalen-1-yl)methylidene]-2-methylalaninato)-zinc), {[ZnL^35^]}_2n_, (**44**), have been synthesized during reaction of zinc nitrate, 2-OH-1-napthaldehyde, and 2-aminoisobutyric acid. The complex crystalizes in monoclinic system. Zinc(II) ions are linked by three Schiff bases, which coordinate the metal centre via an azomethine nitrogen, two carboxylate and two phenolate oxygen atoms. The coordination polyhedron around Zn^II^ adopts a slightly distorted square-pyramidal geometry. Each Schiff base ligand binds three metal ions to form a 2D coordination polymer parallel to the ab plane. (Figure 29). The other coordination polymer, catena-((*μ*-*N*-[(2-oxynaphthalen-1-yl)methylidene]glycinato)-zinc methanol solvate), {[Zn(L^36^)_2_]∙0.33MeOH}_3n_ (**45**), was synthesized in a similar way to the previous one, but glycine was used as the reagent instead of 2-aminoisobutyric acid. This complex crystalizes in the trigonal system, where zinc(II) ions are coordinated in similar way to {[ZnL^35^]}_2n_, i.e., by an azomethine nitrogen, and two carboxylate and two phenolate oxygen atoms of three Schiff base ligands. A different coordination mode of the Schiff base **L^36^** (*anti*, *anti-η*^1^:*η*^1^:*μ*) than that observed for **L^35^** (*syn*, *anti-η*^1^:*η*^1^:*μ*) is probably responsible for the different dimensionality, i.e., 3D framework with channels running along the *c*-axis (Figure 30). Both coordination polymers emit light at a wavelength of around 435 nm, and such luminescent materials can be used as sensors [89].

**Figure 31:**
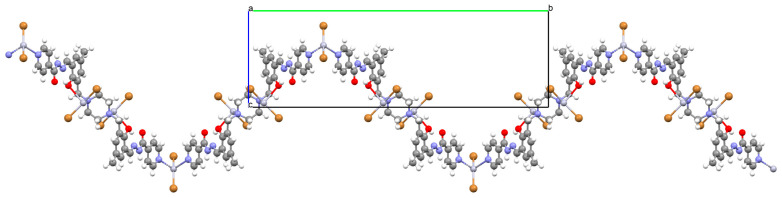
Fragment of crystal structure showing formation of a zig-zag chain of **46** (Table 2) viewed along the *a*-axis [33,90,93].

**Figure 32:**
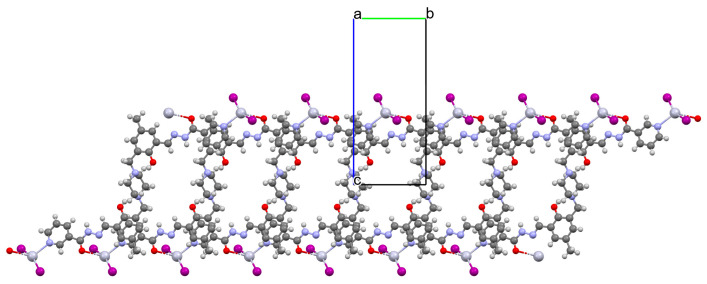
Fragment of crystal structure showing formation of a 1D looped chain of **47** (Table 2) viewed along the *a*-axis [33,90,93]. Solvent molecules are omitted for clarity.

Huang and co-workers investigated complexes of two bispyridyl acylhydrazone Schiff bases (**L^37^** and **L^38^**) with Hg^II^ ion. The first coordination polymer, {[Hg_3_L^37^Br_6_]⋅4DMF}_n_ (**46**) crystalizes in a monoclinic system, where each mercury(II) ion is four-coordinated, and polyhedrons around the metal centres are distorted tetrahedrons. There are two types of mercury(II) ions. The first one is linked by two pyridyl nitrogen atoms from Schiff bases and two bromide ions. The second one is surrounded by two bromide ions, a piperazine nitrogen, and a phenolic oxygen atom of the Schiff base. The bridging nature of the Schiff base leads to the formation of zig-zag chains along the *b*-axis (Figure 31). The second coordination polymer, {[Hg_2_(L^38^)_2_I_4_]⋅2DMF}_n_ (**47**), also crystalizes in a monoclinic system. In this compound, there is only one type of four-coordinated mercury(II) ion, which is linked by a pyridyl nitrogen atom and a phenoxide oxygen atom from two Schiff bases and two iodine ions. The coordination environment around metal ions is also a distorted tetrahedral geometry. This way of binding of metal ions by the *N*,*O*-donor ligand leads to the formation of a 1D looped chain structure (Figure 32). The adsorption properties of the compounds have been studied with respect to molecules such as CH_3_OH, N_2_, H_2_, O_2_, and CO_2_. These compounds show selective and good absorption towards methanol vapour when mixed with other gasses, which may allow them to be used in gas storage and separation [90].

**Figure 33:**
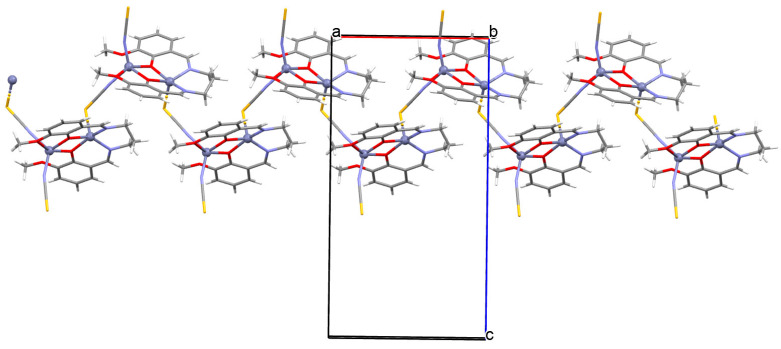
Structure of **48** (Table 2) showing formation of 1D coordination polymer viewed along the *b*-axis [33,91,93].

[Zn_2_L^39^(*μ*_1,3_-SCN)(*η*^1^SCN)]_n_ (**48**) 1D chain thiocyanato bridged coordination polymer (Figure 33) was synthesized during reaction of a *N*,*O*-donor Schiff base (**L^39^** *N*,*N*′-bis(3-methoxysalicylidenimino)-1,3-daminopropane), Zn^II^, and SCN^−^ in the methanol solution. **48** crystallizes in the orthorhombic system. The coordination number of zinc ions is equal to 6 and 5, respectively, with the distorted octahedral (with two phenolate O, two methoxy O, and two thiocyanato N atoms) and the distorted square pyramidal geometry (with two phenoxido O, two imine N atoms in equatorial plane, and one thiocyanato S atom in the axial plane) around the metal centres. Compound **48** shows strong antimicrobial efficacy against some both Gram-positive and Gram-negative strains, whereas the photoluminescent investigation confirmed its credential as a potential photoactive candidate [91].

**Figure 34:**
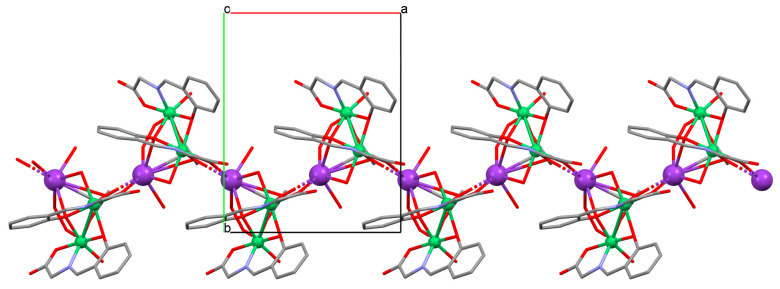
One-dimensional coordination polymer of **49** (Table 2) viewed along the *b*-axis [33,92,93].

The polymeric chain ion {[Ni_2_K(L^40^)_2_Ac(H_2_O)_2_MeOH]·H_2_O}_n_ (**49**) coordination polymer (Figure 34) was derived from a tridentate Schiff base and Ni^II^ in the methanol solution. **49** crystallizes in the monoclinic system. In the crystal, each Ni^II^ centre is six-coordinated and has a distorted octahedral geometry. Compound **49** may be applied as an electrocatalyst in fuel cells using H_2_O_2_ as an oxidant [92].

## 5. Conclusions

The synthesis, crystal structures, and selected promising properties of coordination polymers for potential applications are presented in this paper. The review may be of interest to scientists, who can read about the latest developments in the above-mentioned aspects of coordination polymers with *N*,*O*-donor Schiff base ligands. Single crystal X-ray diffraction analysis revealed that the topology of the coordination polymers is influenced by the differences in coordination modes and conformations of the *N*,*O*-donor Schiff base ligands. These compounds are mainly obtained as 1D chains due to steric effect of ligands used as well as they are built by bridging mononuclear or polynucelear units by additional linkers. The 2D layers or 3D frameworks are rarely formed.

**Figure 35:**
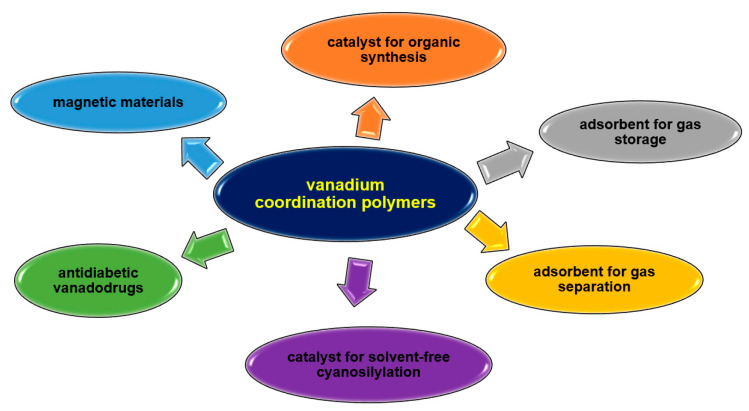
Some potential applications of vanadium coordination polymers.

**Figure 36:**
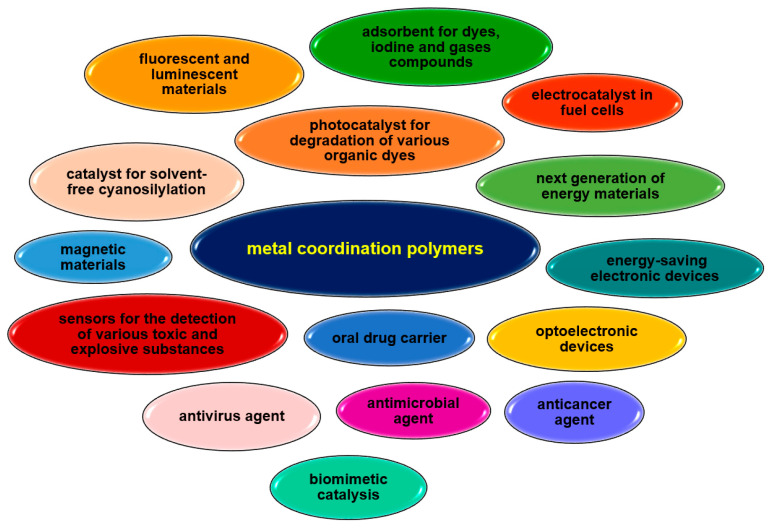
Some potential applications of selected metal ions coordination polymers.

Their unique properties, straightforward synthesis often following with green chemistry rules, and easy availability of reagents are increasing the interest of researchers. Vanadium complexes are mainly under investigation for their use as catalysts in organic synthesis or for their biological or sorption properties (Figure 35). V-CPs highly stable polymeric structures help maintain the structural feature required for catalytic activity during the reaction. The vanadium coordination polymers are mainly studied as catalysts in oxidation reactions, e.g., cyclohexene, trans-stilbene, styrene, isoeugenol, pyrogallol, but can also be used in epoxidation (e.g., alkene, cycloalkene) or cyanation of aldehyde processes. The sorption properties of vanadium polymers are also proving useful. Some complexes are being investigated for their ability to absorb small molecules such as H_2_ and CO_2_, which can be used in gas storage and separation. Coordination polymers of other metal ions also have a much wider range of applications due to their different properties, as shown in Figure 36. These compounds are mainly tested for their biological activity, e.g., antiviral, antimicrobial, anticancer. Their catalytic properties, not only in organic synthesis but also as photocatalysts or electrocatalysts, are of great interest. In recent years, there has been growing interest in the development of metals coordination polymers for use in new technologies, such as energy and electronic materials. Similarly to V-CPs, the coordination polymers of other metals are characterized by a good sorption capacity not only for small molecules such as CH_3_OH, N_2_, H_2_, O_2_, and CO_2_ but also for larger molecules such as dyes.

## Figures and Tables

**Table 1:** Vanadium coordination polymers—scheme and conditions of their synthesis.

Scheme of the Complex	Reagents/M:L Ratio	Conditions/Solvents	Ref.
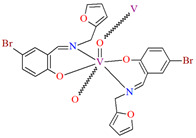 **1**	5-bromo-2-hydroxybenzyl-2-furyl(methyl)imine, VO(acac)_2_ 1:1	the reaction was carried in methanol in reflux conditions MeOH	[66]
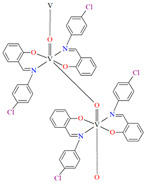 **2**	*bis*[*N*-(4-chlorophenyl) salicylideneaminato], VOSO_4_ 1:2	MeOH	[67]
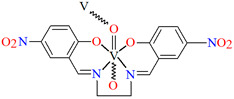 **3**	*N*,*N′*-di-5-nitrosalicylidene-1, 2-ethanediamine), vanadyl sulphate pyridine 1:1	a hot solution was stirred at 130 °C for 1 h MeOH	[68]
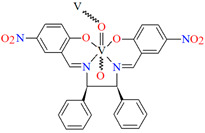 **4**	*N*,*N′*-di-5-nitrosalicylidene-(*R*,*S*)(*S*,*R*)-1,2diphenyl-1,2-ethanediamine), vanadyl sulphate pyridine 1:1	a hot solution was stirred at 130 °C for 1 h MeOH	[68]
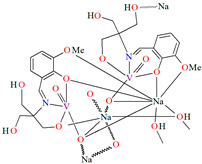 **5**	*o*-vanillin, tromethamine VO(acac)_2_, NaOH 1:1	the resulting solution was refluxed for 2 h at 90 °C under continuous stirring and then cooled to room temperature MeOH	[69]
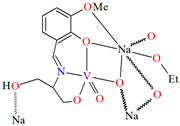 **6**	*o*-vanillin, 2-amino-1,3-propanediol, VO(acac)_2_, NaOH 1:1	the resulting solution was refluxed for 2 h at 90 °C under continuous stirring and then cooled to room temperature EtOH	[69]
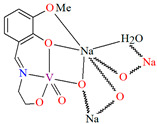 **7**	*o*-vanillin, ethanolamine, VO(acac)_2_, NaOH 1:1	the resulting solution was refluxed for 2 h at 90 °C under continuous stirring and then cooled to room temperature EtOH	[69]
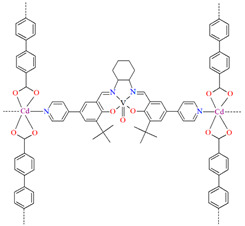 **8**	((*R*,*R*)-(–)-1,2-cyclohexanediamino-*N*,*N*’bis(3-tert-butyl-5-(4-pyridyl)salicylidene), biphenyl-4,4′-dicarboxylic acid, VOSO_4_, Cd(NO_3_)_2_·4H_2_O	synthesis under solvothermal conditions at 100 °C DMF/EtOH/H_2_O	[70]
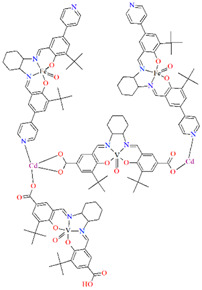 **9**	Cd(NO_3_)_2_∙6H_2_O, VO(L^9^), FeL^8^(OAc)	a solution was stirred at 80 °C DMF/MeOH	[71]
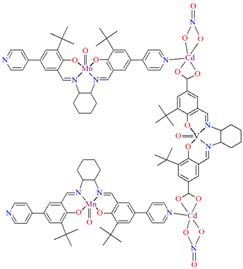 **10**	Cd(NO_3_)_2_∙6H_2_O, VO(L^9^), MnL^8^Cl	a solution was stirred at 80 °C DMF/EtOH	[71]
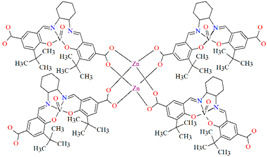 **11**	salen dicarboxylate, VO(acac)_2_, ZnI_2_	a solution was heating at 85 °C DMA/MeOH/H_2_O	[72]
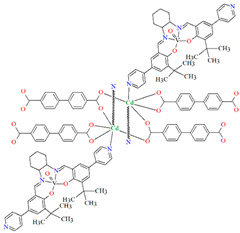 **12**	salen dicarboxylate, VO(acac)_2_, CdI_2_	a solution was heating at 85 °C DMA/MeOH	[72]
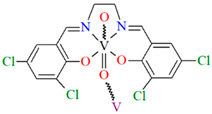 **13**	*N*,*N′*-ethylenebis(3,5-dihalosalicylideneaminate), pyridine oxovanadium(IV) sulphate	a slow diffusion method was applied by contacting two dilute solutions containing reagents	[73]
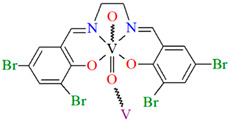 **14**	*N*,*N′*-ethylenebis(3,5-dihalosalicylideneaminate), pyridine oxovanadium(IV) sulphate	a slow diffusion method was applied by contacting two dilute solutions containing reagents	[73]
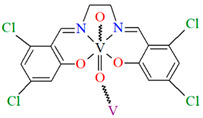 **15**	*N*,*N′*-ethylenebis(4,6-dihalosalicylideneaminate), pyridine oxovanadium(IV) sulphate	a slow diffusion method was applied by contacting two dilute solutions containing reagents	[73]
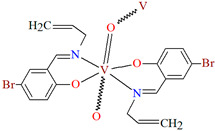 **16**	allylamine, 5-bromo salicylaldehyde, VO(acac)_2_ 1:1	the reaction solution was heated at reflux condition with constant magnetically stirring for 3 h MeOH	[74]
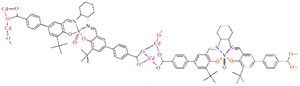 **17**	(1*R*,2*R*)-[VO(L^14^)], Cd(NO_3_)_2_∙6H_2_O 1:1	the mixture was heated at 100 °C for 12 h DMF	[75]
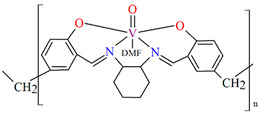 **18**	**L^15^**, VO(acac)_2_ 1:1	heating on a water bath MeOH/DMF	[76]
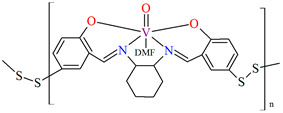 **19**	**L^16^**, VO(acac)_2_ 1:1	heating on a water bath MeOH/DMF	[76]
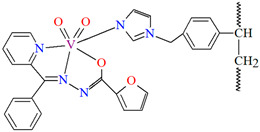 **20**	**L^17^**, VO(acac)_2_ 1:1 PS-im (imidazolo- methylpolystyrene)	refluxed under stirring in an oil bath for 2 h MeOH	[77]
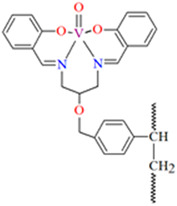 **21**	**L^18^**, VOSO_4_·5H_2_O, VO(acac)_2_ 1:1 PS-Cl (choloromethylated polystyrene)	the reaction mixture was refluxed, complex was dissolved in hot DMSO MeOH/DMSO	[78]
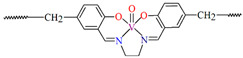 **22**	**L^19^**, VO(acac)_2_ 1:1	the reaction mixture was digested on a water bath at ca. 90 °C for 4 h. MeOH/DMF	[79]
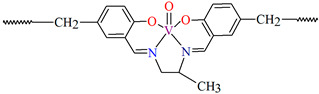 **23**	**L^20^**, VO(acac)_2_ 1:1	the reaction mixture was digested on a water bath at ca. 90 °C for 4 h. MeOH/DMF	[79]
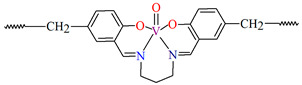 **24**	**L^21^**, VO(acac)_2_ 1:1	the reaction mixture was digested on a water bath at ca. 90 °C for 4 h. MeOH/DMF	[79]

**Table 2:** Coordination polymers of selected metal ions—scheme and conditions of their synthesis.

Scheme of the Complex	Reagents/M:L Ratio	Conditions/Solvents	Ref.
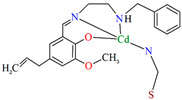 **25**	4-allyl-2-(((2-(benzylamino)ethyl)imino) methyl)-6-methoxyphenol, Cd(NO_3_)_2_·4H_2_O, NaSCN 1:1	the reaction mixture was stirred at room temperature for 4 h MeOH	[80]
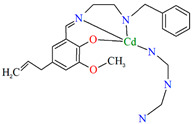 **26**	4-allyl-2-(((2-(benzylamino)ethyl)imino) methyl)-6-methoxyphenol, Cd(NO_3_)_2_·4H_2_O, NaN(CN)_2_ 1:1	the reaction mixture was stirred at room temperature for 4 h MeOH	[80]
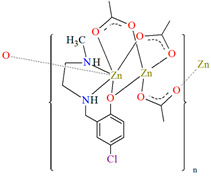 **27**	4-chloro-2-(((2-(methylamino)ethyl)amino)methyl)phenol, Zn(OAc)_2_·2H_2_O 2:1	the reaction mixture was constant stirred MeOH	[81]
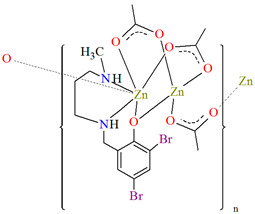 **28**	2,4-dibromo-6-(((3-(methylamino)propyl)amino)methyl) phenol, Zn(OAc)_2_·2H_2_O 2:1	the reaction mixture was constant stirred MeOH	[81]
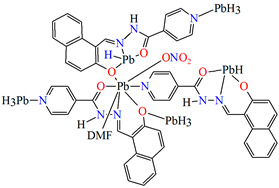 **29**	(2-hydroxynaphthalen-1-ylmethylene)-hydrazide, Pb(NO_3_)_2_ 1:1	the reaction mixture was constant stirred MeOH/DMF/H_2_O	[1]
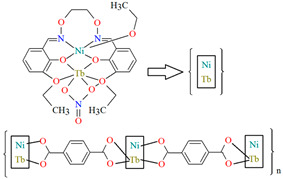 **30**	**L^26^**, terephthalic acid, Ni(OAc)_2_·4H_2_O, Tb(NO_3_)_3_·6H_2_O 1:1	the reaction mixture was continuously stirring for 20 min EtOH/DMF/CHCl_3_	[82]
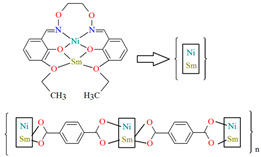 **31**	**L^26^**, terephthalic acid, Ni(OAc)_2_·4H_2_O, Sm(NO_3_)_3_·6H_2_O 1:1	the reaction mixture was continuously stirring for 20 min EtOH/DMF/CHCl_3_	[82]
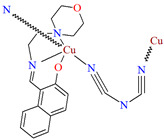 **32**	(1-(2-(morpholino-ethylimino)methyl)naphthalen-2-ol), Cu(OAc)_2_·H_2_O, NaN(CN)_2_ 1:1	the reaction mixture was constant stirring for 2 h MeOH	[83]
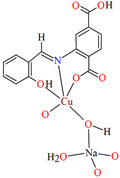 **33**	2-[(*E*)-(2-hydroxy-phenyl)methyleneamino]terephthalic acid, Cu(NO_3_)_2_·3H_2_O NaOH 1:1	The reaction mixture was refluxed for 1 h. MeOH/H_2_O	[84]
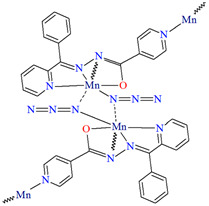 **34**	(*E*)-*N*′-(phenyl(pyridin-2-yl)methylene) Isonicotinhydrazide, MnCl_2_·4H_2_O, NaN_3_ 1.1:1	complex was synthesized using the thermal gradient method in a branched tube in an oil bath at 60 °C MeOH	[85]
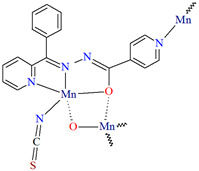 **35**	(*E*)-*N*′-(phenyl(pyridin-2-yl)methylene) Isonicotinhydrazide, MnCl_2_·4H_2_O KSCN 1.1:1	complex was synthesized using the thermal gradient method in a branched tube in an oil bath at 60 °C MeOH	[85]
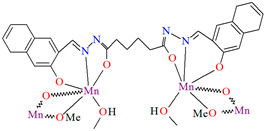 **36**	bis[(2-hydroxynaphthalen-1-yl)methylene]-adipohydrazide, MnCl_2_·4H_2_O 2:1	MeOH	[86]
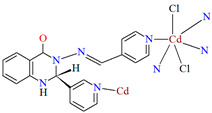 **37**	2-amino-*N*′-(pyridin-4-ylmethylene)-benzohydrazide, CdCl_2_ 2:1	the reaction was performed at room temperature for 1 h DMF/MeOH	[87]
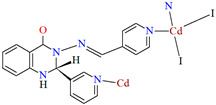 **38**	2-amino-*N*′-(pyridin-4-ylmethylene)-benzohydrazide, CdI_2_ 1:1	the reaction was performed at room temperature for 1 h DMF/MeOH	[87]
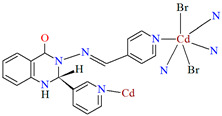 **39**	2-amino-*N*′-(pyridin-4-ylmethylene)-benzohydrazide, CdBr_2_·4H_2_O 2:1	the reaction was performed at room temperature for 1 h DMF/MeOH	[87]
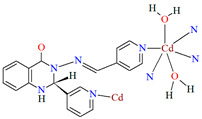 **40**	2-amino-*N*′-(pyridin-4-ylmethylene)-benzohydrazide, Cd(NO_3_)_2_·4H_2_O 2:1	the reaction was performed at room temperature for 1 h MeOH	[87]
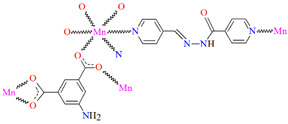 **41**	pyridine-4-carboxaldehydeisonicotinoylhydrazine, 5-aminoisophthalic acid, MnCl_2_·4H_2_O 1:1	DMF/MeOH	[88]
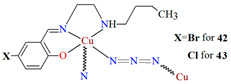 **42**, **43**	*N*-butyl-*N*-(5-bromosalcylidine)ethane-1, 2-diamine or *N*-butyl-*N*-(5-chlorosalcylidine)ethane-1, 2-diamine, Cu(OAc)_2_·H_2_O, NaN_3_ 1:1	A methanolic solution of ligand and Cu^2+^ was refluxed for 1 h. Further aqueous solution of NaN_3_ was added and refluxed for another 2 h. MeOH/H_2_O	[4]
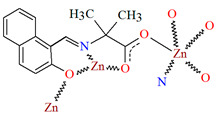 **44**	2-OH-1-napthaldehyde-2-aminoisobutyric acid, Zn(NO_3_)_2_·4H_2_O 1:1	the reaction was performed in autoclaves at 120 °C for 12 h MeOH	[89]
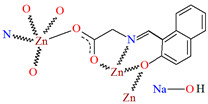 **45**	2-OH-1-napthaldehyde-glycine acid, Zn(NO_3_)_2_·4H_2_O 1:1	the reaction was performed in autoclaves at 120 °C for 12 h MeOH	[89]
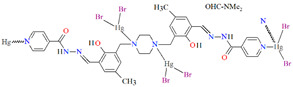 **46**	Dialdehyde, isonicotinic hydrazide, HgBr_2_ 3:1	The mixture was stirred with heating for 1 h DMF/MeOH	[90]
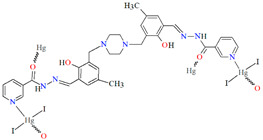 **47**	Dialdehyde, isonicotinic hydrazide, HgI_2_ 2:1	The mixture was stirred with heating for 1 h DMF/MeOH	[90]
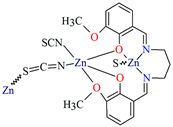 **48**	*N*,*N*′-bis(3-methoxysalicylidenimino)-1,3-daminopropane, Zn(OAc)_2_·2H_2_O, KSCN 1:1	in presence of KSCN, constant stirring for 2.5 h MeOH	[91]
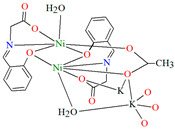 **49**	Glycine, potassium hydroxide, salicylaldehyde, Ni(OAc)_2_·4H_2_O 1:1	a solution of metal ion was slowly added to the solution of the Schiff base MeOH	[92]

## Data Availability

No new data were created or analyzed in this study.
